# Dynamics of Neuronal and Astrocytic Energy Molecules in Epilepsy

**DOI:** 10.1111/jnc.70044

**Published:** 2025-03-20

**Authors:** Kota Furukawa, Yoko Ikoma, Yusuke Niino, Yuichi Hiraoka, Kohichi Tanaka, Atsushi Miyawaki, Johannes Hirrlinger, Ko Matsui

**Affiliations:** ^1^ Super‐network Brain Physiology Graduate School of Life Sciences, Tohoku University Sendai Japan; ^2^ Laboratory for Cell Function Dynamics RIKEN Center for Brain Science Wako‐City Japan; ^3^ Laboratory of Molecular Neuroscience Medical Research Institute (MRI), Tokyo Medical and Dental University (TMDU), Tokyo Institute of Technology Tokyo Japan; ^4^ Laboratory of Genome Editing for Biomedical Research Medical Research Institute, Tokyo Medical and Dental University (TMDU), Tokyo Institute of Technology Tokyo Japan; ^5^ Biotechnological Optics Research Team RIKEN Center for Advanced Photonics Wako‐City Japan; ^6^ Carl‐Ludwig‐Institute for Physiology, Faculty of Medicine Leipzig University Leipzig Germany; ^7^ Department of Neurogenetics Max‐Planck‐Institute for Multidisciplinary Sciences Göttingen Germany; ^8^ Super‐network Brain Physiology, Graduate School of Medicine Tohoku University Sendai Japan

**Keywords:** astrocyte, ATP, blood vessels, epilepsy, fiber photometry, pyruvate

## Abstract

The dynamics of energy molecules in the mouse brain during metabolic challenges induced by epileptic seizures were examined. A transgenic mouse line expressing a fluorescence resonance energy transfer (FRET)‐based adenosine triphosphate (ATP) sensor, selectively expressed in the cytosol of neurons, was used. An optical fiber was inserted into the hippocampus, and changes in cytosolic ATP concentration were estimated using the fiber photometry method. To induce epileptic neuronal hyperactivity, a train of electrical stimuli was delivered to a bipolar electrode placed alongside the optical fiber. Although maintaining a steady cytosolic ATP concentration is crucial for cell survival, a single episode of epileptic neuronal hyperactivity drastically reduced neuronal ATP levels. Interestingly, the magnitude of ATP reduction did not increase with the exacerbation of epilepsy, but rather decreased. This suggests that the primary consumption of ATP during epileptic neuronal hyperactivity may not be solely directed toward restoring the Na^+^ and K^+^ ionic imbalance caused by action potential bursts. Cytosolic ATP concentration reflects the balance between supply and consumption. To investigate the metabolic flux leading to neuronal ATP production, a new FRET‐based pyruvate sensor was developed and selectively expressed in the cytosol of astrocytes in transgenic mice. Upon epileptic neuronal hyperactivity, an increase in astrocytic pyruvate concentration was observed. Changes in the supply of energy molecules, such as glucose and oxygen, due to blood vessel constriction or dilation, as well as metabolic alterations in astrocyte function, may contribute to cytosolic ATP dynamics in neurons.

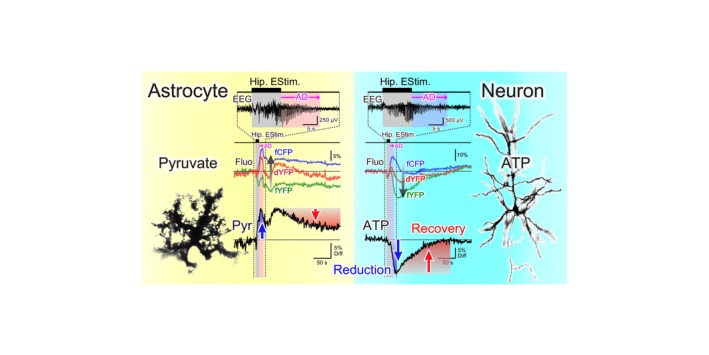

## Graphical abstract

The dynamics of energy molecules in the mouse brain during metabolic challenges induced by epileptic seizures were studied using transgenic mice with FRET‐based pyruvate or ATP sensors selectively expressed in astrocytes or neurons, respectively. In response to epileptic neuronal hyperactivity, astrocytic cytosolic pyruvate levels increased, while neuronal cytosolic ATP levels decreased. The magnitude of neuronal ATP reduction diminished following the exacerbation of epilepsy, suggesting alterations in the transfer of energy‐generating substances from astrocytes to neurons.

**Abbreviations**
AD: after‐discharge
AED: anti‐epileptic drug
ANLS: astrocyte‐neuron lactate shuttle
ATP: adenosine triphosphate
BBV: brain blood volume
dYFP: fluorescence emission of yellow fluorescence protein by direct excitation of YFP
ECoG: electrocorticography
EEG: electroencephalogram
fCFP: fluorescence emission of cyan fluorescence protein by direct excitation of CFP (minus the FRET‐mediated loss of emission of CFP)
FRET: fluorescence resonance energy transfer
fYFP: fluorescence emission of YFP by excitation of CFP and FRET‐mediated excitation of YFP
MCT: monocarboxylate transporter
NBC: Na^+^‐bicarbonate cotransporter
PMT: photomultiplier tube
SUDEP: sudden unexpected death in epilepsy
TCA: tricarboxylic acid

## 1 Introduction

Although the brain accounts for only 2% of total body weight, it consumes 20% of the glucose circulating in the blood (Sokoloff et al. 1977). This energy is efficiently utilized to perform complex information processing, which in some aspects can be considered comparable to that of modern computers. However, a loss of blood flow to the brain for as little as approximately 10 s can lead to loss of consciousness in humans. Therefore, the brain's energy supply is likely finely tuned to balance energy consumption, ensuring sufficient neuronal activity to maintain proper cognitive function.

Intracellular ATP serves as an essential energy‐supplying molecule necessary for the functioning and survival of all cells in living organisms. Consequently, multiple mechanisms likely exist to maintain a steady and constant level of ATP in cells, and it is generally not assumed that ATP concentrations dynamically fluctuate in healthy, living cells. Cytosolic ATP levels are determined by the balance between consumption and supply. In neurons, a significant portion of ATP is thought to be consumed by Na^+^/K^+^‐ATPase (Harris et al. 2012). During action potential firing and synaptic activity, extracellular Na^+^ flows into neurons while intracellular K^+^ flows out. ATP is used to pump Na^+^ out and bring K^+^ back in by Na^+/^K^+^‐ATPase, thereby restoring ionic balance. As a result, bursts of neuronal activity are likely to increase ATP consumption; however, the reduction in ATP is most likely counteracted by increased ATP synthesis (Baeza‐Lehnert et al. 2019). Neuronal cytosolic ATP is also required for neurotransmitter synthesis, packaging them into synaptic vesicles, regulating synaptic vesicle cycling, expressing and trafficking receptors and ion channels, and modulating synaptic plasticity. Therefore, the availability of ATP in neurons can directly impact the brain's information‐processing capacity.

With the supply of glucose and oxygen, cells can efficiently produce ATP. Astrocytes contact both blood vessels and neurons, whereas neurons lack direct physical contact with blood vessels. Therefore, glucose from the blood is taken up by astrocytes and converted into pyruvate through glycolysis. In astrocytes, pyruvate is then converted into lactate by lactate dehydrogenase (LDH) and released into the extracellular space via the low‐affinity monocarboxylate transporters (MCT) 1 and 4. Neurons take up this lactate through the high‐affinity MCT2. This process of energy substrate transfer from astrocytes to neurons is called the astrocyte–neuron lactate shuttle (ANLS) (Pellerin and Magistretti 1994; Mächler et al. 2016). Since MCTs can also transport pyruvate, it remains debated whether astrocytes deliver pyruvate or lactate to neurons. In either case, it is generally assumed that monocarboxylates are delivered to neurons, where they are converted into pyruvate. The pyruvate is then taken up into neuronal mitochondria, activating the tricarboxylic acid (TCA) cycle, which produces nicotinamide adenine dinucleotide (NADH). This, in turn, generates a substantial amount of ATP via the electron transport chain and oxidative phosphorylation. Although neurons can also directly take up glucose and neuronal glycolysis has been shown to be the primary source of energy production in neurons in certain situations (Díaz‐García et al. 2017; Díaz‐García and Yellen 2019), the ANLS likely still plays a role in delivering energy substrates to neurons in other circumstances. Inhibition of ANLS or reduction of lactate supply to neurons can impair long‐term memory and cause severe delayed neuronal damage upon ischemia (Schurr et al. 2001; Suzuki et al. 2011).

In this study, we challenged the mouse brain with metabolic stress via epilepsy to examine how effectively the neuron‐metabolic system can maintain its stability. In rodents, electrical stimulation of the limbic system has been observed to elicit characteristic electroencephalogram (EEG) oscillations known as after‐discharge (AD), which persist even after the cessation of stimulation (Goddard et al. 1969). This AD exhibits similarities to the EEG patterns seen during spontaneous seizures in human epilepsy patients and thus serves as a model for studying epilepsy in experimental animals (Araki et al. 2024; Stern et al. 2024). With repeated hippocampal stimulation, the duration and intensity of epileptic neuronal hyperactivity increased through a process known as kindling (Shimoda et al. 2022; Ikoma, Sasaki, et al. 2023). We used transgenic mice expressing a FRET‐based sensor for ATP in the neuronal cytosol (Thy1‐ATeam mice; Trevisiol et al. 2017). By measuring fluorescence intensity, we monitored neuronal cytosolic ATP dynamics associated with epileptic neuronal hyperactivity. Local blood volume changes were visualized using Texas Red dextran, a fluorescent dye injected into the tail vein (Asano et al. 2024; Tan et al. 2024). Pyruvate concentration changes were assessed using another transgenic mouse line expressing a FRET‐based pyruvate sensor (PYRS) specifically in astrocytes (Mlc1‐tTA::tetO‐PYRS mice).

Epilepsy is estimated to affect 68 million people worldwide (Ngugi et al. 2010). Anti‐epileptic drugs (AEDs) are typically designed to target ion channels and transporters, aiming to reduce excitation or enhance inhibitory neurotransmission. While new drugs are continuously being developed, about one‐third of epilepsy patients remain refractory to AEDs (Rogawski et al. 2016; Chen et al. 2018; Kwan and Brodie 2000). For patients with intractable epilepsy, the ketogenic diet has proven effective in some cases. The ketogenic diet forces the body to use fat as an energy source instead of glucose. In recent years, increasing attention has been given to targeting metabolism as a way to control epileptic seizures (Lutas and Yellen 2013; Cervenka et al. 2021). By better understanding the metabolic processes underlying epilepsy pathology, novel treatment strategies may be developed (Sada et al. 2015; Düking et al. 2022). We also believe that studying how the neuron –metabolic system operates under pathological conditions could lead to insights into how the brain efficiently performs complex information processing while using only the minimal energy required, comparable to lighting a dim light bulb.

## 2 Materials and Methods

### 2.1 Animals

All experiments were conducted in accordance with the Regulations for Animal Experiments and Related Activities at Tohoku University (2019LsA‐017). Efforts were made to minimize animal suffering and reduce the number of animals used. The mice were kept at 24°C–26°C with a 12‐h light–dark cycle, and they had *ad libitum* access to regular chow and water. After weaning, the mice were housed with littermates of the same sex to promote environmental enrichment. Since epileptic seizure characteristics and epileptogenesis differ between males and females (Scharfman and MacLusky 2014), only male mice older than 15 weeks were used in this study (total number of mice used, 21). Surgical implantation of electrodes and optical fibers was performed under anesthesia (see below), and butorphanol was used as an analgesic, with its effects lasting for a few hours post‐surgery. To facilitate recovery, animals were housed in a cage placed on a warm plate, which was set to approximately 30°C, for a couple of days. Water was supplied in a gel form to facilitate intake during this phase. Nesting material was placed in the cage for environmental enrichment. Each animal was housed individually to prevent damage to the implanted apparatus from other mice. During the 5‐day post‐surgical recovery period, animals were monitored for health twice daily. If signs of distress or minor weight loss were observed, the animal was immediately euthanized to ensure humane treatment. Initial electrophysiological and fiber photometry tests were conducted to check for any signal detection issues. If any abnormalities were found, the animal was promptly euthanized without further experimentation. After passing these tests, all animals were included in the study, and data collection proceeded without exclusions. In this study, two mice scheduled for kindling experiments died during the recovery period following optical fiber implantation surgery prior to the start of the experiment. Additionally, three animals died due to sudden unexpected death in epilepsy (SUDEP) during the kindling experiment. For euthanization following experiments, animals were placed in a closed chamber with vaporized isoflurane (~1 mL per 1 L of space) to induce an overdose of anesthesia through inhalation. Death was confirmed by the absence of respiration.

*Thy1‐ATeam* transgenic mice were used to monitor cytosolic ATP concentration dynamics in neurons. Details of the transgenic mice are described elsewhere (Trevisiol et al. 2017), but briefly, the expression of the fluorescence resonance energy transfer (FRET)‐based ATP sensor ATeam1.03^YEMK^ (referred to as ATeam in the following, Imamura et al. 2009) was driven by the neuron‐specific *Thy1* promoter. ATeam consists of cyan fluorescence protein (CFP) and yellow fluorescence protein (YFP), which are connected by the *ε* subunit of *Bacillus subtilis* F0F1‐ATP synthase. This *ε* subunit serves as the ATP‐specific binding domain. In the absence of ATP, the extended and flexible conformation of the *ε* subunit separates the two fluorescent proteins, reducing the efficiency of FRET. When ATP is bound, the *ε* subunit retracts, bringing the two fluorescent proteins closer together and thereby increasing FRET efficiency. The apparent dissociation constant for ATeam1.03^YEMK^ is 1.2 mM.

*Mlc1‐tTA::tetO‐PYRS* double‐transgenic mice were used to monitor cytosolic pyruvate concentration dynamics in astrocytes. In *Mlc1‐tTA* transgenic mice, the astrocyte‐specific *Mlc1* promoter drives the expression of the tetracycline transactivator (tTA) (Tanaka et al. 2012). The *tetO‐PYRS* knockin mouse was newly established by inserting a *tetO‐PYRS‐hGHpA* cassette into the 3′ untranslated region of the mouse *Actb* gene using the CRISPR/Cas9‐mediated genome editing technology, as described previously (Aida et al. 2015). The *Mlc1‐tTA* transgenic mice were crossbred with the *tetO‐PYRS* knock‐in mice. In the double‐transgenic mice, tTA, selectively expressed in astrocytes, activates the *tetO* promoter, leading to the expression of the FRET‐based pyruvate sensor, PYRS, in a subpopulation of astrocytes (Kanemaru et al. 2014). PYRS was generated by connecting CFP and YFP to the pyruvate dehydrogenase regulator (PdhR) from *Escherichia coli* . PdhR serves as a domain that binds pyruvate specifically. The ligand binding affects the conformational relationship between CFP and YFP, thereby modulating the FRET efficiency. The gene for PdhR was synthesized with mammalian‐preferred codons and modified for the construction of PYRS. The apparent dissociation constant and the Hill coefficient for pyruvate were calculated to be 26 μM and 1.2, respectively. The nucleotide sequence for the PYRS cDNA has been deposited to the DNA Data Bank of Japan (DDBJ)/European Molecular Biology Laboratory (EMBL)/GenBank databases (accession number LC849112). Similar to previously reported FRET‐based sensors possessing PdhR (San Martín et al. 2014; Chandris et al. 2022), an increase in pyruvate concentrations results in an increase in CFP emission and a decrease in YFP emission. The conformational change that occurs upon the binding of pyruvate to PdhR likely separates the CFP and YFP, leading to a reduction in FRET efficiency.

### 2.2 Surgery

Prior to the surgical implantation of the optical fiber, the mouse was anesthetized with intraperitoneal administration of a combination of three types of mixed anesthesia: 0.75 mg/kg of medetomidine hydrochloride (Domitor; Nippon Zenyaku Kogyo Co. Ltd., Fukushima, Japan), 4 mg/kg of midazolam (Midazolam; Sandoz Inc., Japan), and 5 mg/kg of butorphanol tartrate (Vetorphale; Meiji Seika Pharma Co. Ltd., Tokyo, Japan). The anesthetized mouse was then secured to an auxiliary ear bar and mounted on a stereotaxic frame for the surgery. The coordinates were based on the mouse brain atlas (Paxinos and Franklin 2001). To reverse the effects of medetomidine after surgery, 0.75 mg/kg of atipamezole hydrochloride (Antisedan; Nippon Zenyaku Kogyo Co. Ltd., Tokyo, Japan) was administered. Fiber photometry measurements began at least five days after surgery, while rapid kindling experiments started 1 to 2 days after recording the baseline fluorescence.

After shaving the fur on the head of the anesthetized mouse, the area was disinfected with ethanol, and the scalp was cut to expose the skull. Prior to implanting stainless‐steel screws (M1) for electrophysiological recordings, two cranial holes were drilled above the cerebral cortex and the cerebellum, with each hole's diameter slightly less than 1 mm. A screw with a diameter of 1 mm was inserted into each cranial hole using a fine Phillips head screwdriver until the far end of the screw just touched the brain surface. The screw implanted in the cerebellum served as a common ground, while the screw in the cerebral cortex was used for intracranial electroencephalography (EEG), more specifically, electrocorticography (ECoG) recordings.

Two tungsten electrodes (bare diameter, 127 μm) had their PFA coating (total diameter, 178 μm; cat. no. 796500; AM‐Systems, WA, USA) removed for approximately 0.3 mm from the tip. A glass optical fiber (core diameter, 400 μm, 0.39 NA; cat. no. FT400UMT; Thorlabs) with a ceramic ferrule (external diameter, 2.5 mm) was also prepared; this assembly was created by Kyocera, Kyoto, Japan. The pair of tungsten electrodes was adhered to the base of the ferrule using UV curing resin. This “opto‐rode” was inserted into the dentate gyrus of the hippocampus at coordinates 1.94 mm posterior and 0.9 mm lateral to the bregma, at a depth of 1.8 mm from the cortical surface. The location of the opto‐rode insertion was confirmed post‐experiments in most cases. In 5 out of 11 cases examined, the optical fiber tip protruded well into the dentate gyrus, whereas in 6 cases, it was positioned more superficially in the CA1 region. No consistent differences in fluorescence signals were observed based on the exact location of the optical fiber tip. An electrical current passing through these electrodes was used to stimulate the hippocampus and induce seizures.

The opto‐rode assembly and the stainless‐steel screws were fixed onto the mouse skull with synthetic resin (Super Bond C&B; Sun Medical Company). Lead wires from all four electrodes (two tungsten electrodes in the hippocampus, one screw electrode in the cortex, and one screw electrode in the cerebellum) were soldered to a multi‐male pin connector assembly. To prevent short circuits, the gaps between the wires were filled with instant adhesive (Loctite 454J; Henkel), which served as insulation. Subsequently, the screw electrodes, the opto‐rode, and the pin connector were securely fixed to the dried skull with dental cement, ensuring a stable attachment of the entire apparatus.

Post‐surgery, the mice were housed separately for a recovery period of at least 5 days. After recovery and prior to the experiments, an optical fiber and a multicore electrical cable were connected to the connector assembly fixed on the skull. For recording optical signals, the optical ferrule of the opto‐rode assembly implanted in the hippocampus was connected to another ferrule at the terminal of an optical fiber linked to the fiber photometry apparatus (Lucir, Tsukuba, Japan). The multi‐male pin connector assembly on the skull was connected to a multicore electrical cable with a multi‐female pin connector assembly at its terminal. The other end of the multicore cable was loosened, and each individual core was soldered to separate pins. The pins from the cable cores connected to the screw electrodes in the cortex and cerebellum were routed to a bioelectrical amplifier (cat. no. AB‐611J; Nihon Kohden, Tokyo, Japan), where the signals were amplified and filtered between 0.5 Hz and 1 kHz. The cortical EEG was digitally recorded at a sampling frequency of 2 kHz using Spike2 7.20 software (Cambridge Electronic Design Limited, UK), which controlled the Micro1401‐3 plus ADC12 top box (CED, Cambridge, UK). The pins from the cable cores connected to the stimulating electrodes implanted in the hippocampus were linked to a constant‐current isolated stimulator (cat. no. DS3; Digitimer, Hertfordshire, UK). The electrical stimulation pattern was created using Master‐8 (A.M.P.I., Jerusalem, Israel).

### 2.3 Rapid Hippocampal Kindling Protocol

A train of electrical stimulation (0.04 mA in amplitude, 1 ms pulses, 50 Hz frequency, 10 s duration) was delivered between the two electrodes inserted into the hippocampus using a constant‐current isolated stimulator (DS3). These train stimulations were applied 12 times a day from 21:00 to 8:00 (Zeitgeber Time 12.5 to 23.5) during the dark period, with one‐hour intervals for 3 to 5 consecutive days in most cases. As mice are nocturnal animals, they are more active during the nighttime. Delivering kindling stimuli exclusively at night causes less sleep deprivation compared to stimulating during the daytime. In rare instances, electrical stimulation had to be halted for up to one week, after which the kindling protocol continued. However, even in such cases, the seizure scale (described below) and the kindling level were maintained before and after the long pause, allowing the kindling to resume. Behavioral responses to the epileptic kindling stimuli were monitored using an infrared video recording system.

ADs are oscillatory waveforms observed in the EEG following the cessation of the train stimuli, which last typically for 5 to 20 s in the case of initial evoked responses. These initial train stimulations usually did not evoke a behavioral seizure response (Stage A; see below). The rapid kindling protocol employed in this study was similar to those used in previous research (Lothman and Williamson 1993; Etemadi et al. 2015). Following multiple episodes of train stimulation, the duration of the ADs gradually increased, and the EEG waveform became more complex, exhibiting multiple frequency components during a single episode. The behavioral seizure responses observed in our study were slightly different from those reported in rats during kindling (Racine 1972). Consequently, we devised a new seizure scale for our mouse experiments as follows: Stage A—noticing of an incident; Stage B—tail lifting; Stage C—wild running; SUDEP—sudden unexpected death in epilepsy (Ikoma, Sasaki, et al. 2023). With repeated train stimulations, the progression from Stage A to Stage C typically occurred within 3 to 5 consecutive days.

### 2.4 Fiber Photometry

A custom‐made optical apparatus for fiber photometry (Lucir) was utilized in this study. Excitation lights were delivered from the SPECTRA X Light Engine (Lumencor, OR, USA). An optical fiber (core diameter: 400 μm, 0.39 NA; cat. no. FT400UMT; Thorlabs) connected the fiber photometry apparatus to the implanted opto‐rode terminal on the animal's skull. The terminal end of the optical fiber at the animal's end was covered with a 2.5 mm external diameter ceramic ferrule (cat. no. CF440). The opto‐rode placed on the skull also had the same diameter ferrule (assembled by Kyocera). These two ferrules were connected using a mating sleeve (cat. no. ADAF1; Thorlabs). The excitation light was transmitted through the optical fiber from the fiber photometry apparatus to the animal, while the emitted light returned through the optical fiber, reaching the fiber photometry apparatus. The emitted light was detected using two photomultiplier tubes (PMTs) (cat. no. PMTH‐S1‐CR316‐02; Zolix Instruments Co. Ltd., China) equipped with current‐to‐voltage amplifiers (cat. no. HVC1800; Zolix). The electronic signals from the PMTs were digitized using the Micro1401‐3 plus ADC12 top box (CED) and recorded with Spike2 software (CED) at a 2 kHz sampling frequency, alongside the electrophysiological recordings.

Thy1‐ATeam and PYRS fluorescence recording configuration: The fluorescence recording setup utilized two wavelength excitations generated by the SPECTRA X Light Engine: Blue (~440 nm) and Teal (~510 nm). A multi‐bandpass filter (cat. no. 89402m; Chroma, VT, USA) ensured the designated wavelengths for the excitation of cyan fluorescence protein (CFP) and yellow fluorescence protein (YFP), respectively. Specifically, the light for directly exciting YFP was delivered via the 519 nm/26 nm (FWHM) band of the multi‐bandpass filter. This configuration allowed negligible light with a wavelength below 500 nm to pass through when the Teal excitation light was generated. Since the excitation wavelength for CFP is below 490 nm, it can be assumed that the excitation of CFP is minimal when using light directly aimed at exciting YFP. A multi‐band beamsplitter (cat. no. ZT442/514rpc; Chroma) was employed to direct multiple excitation lights to the mice while allowing the emission lights from both CFP and YFP to pass through for detection. The excitation power at the tip of the bare optical fiber inserted into the brain was approximately 0.5 mW for the Blue light and ~0.07 mW for the Teal light. The emitted light from the animal's brain traveled back through the optical fiber and the multi‐band beamsplitter. The two‐wavelength emissions were subsequently separated using another dichroic mirror (cat. no. T495lpxr; Chroma), with CFP and YFP detected through their respective bandpass filters (cat. no. ET473/24m and cat. no. ET560/50m; Chroma).

To ensure consistent readings, the excitation power of the two LEDs and the driving voltages of the two PMTs were adjusted so that the output from the PMTs was approximately 2 V across all excitation and emission combinations. Despite careful design, some bleed‐through of strong excitation light through the emission filter was observed. To account for this, the tip of the optical fiber was covered with black aluminum foil to calculate the background PMT output level, which was subsequently subtracted from the recorded traces.

The two wavelengths of excitation light were sequentially delivered in 20 ms pulses. Specifically, the Blue excitation light (~440 nm) was illuminated for 20 ms, followed by a 10 ms interval before the Teal excitation light (~510 nm) was illuminated for another 20 ms. After an interval of 950 ms, the same sequence would start again. This ‘gallop’ mode sequence ensured near‐simultaneous recording of fluorescence signals from the two excitation wavelengths while maintaining a relatively low sampling frequency of 1 Hz. Although the outputs from the two PMTs were continuously recorded, the output waveform within each 20 ms pulse was averaged, resulting in an actual sampling frequency of 1 Hz for each excitation light. The signals from the two PMTs were digitized and recorded in conjunction with the electrophysiological recordings. Excitation pulses were delivered and emission recorded for 40 min out of each hour, with the remainder of the hour primarily dedicated to saving the recorded data. Prior to the kindling experiments, at least 24 h were spent collecting baseline fluorescence fluctuations.

ATeam and Texas Red recording configuration: To visualize local brain blood volume dynamics directly, Texas Red dextran (70 000 MW, Invitrogen) was injected via the tail vein at a dosage of 25 mg/kg in 0.1 mL saline. A triple wavelength excitation was employed: Blue light (~440 nm) for the excitation of CFP (fCFP) and for FRET‐mediated excitation of YFP (fYFP), Teal light (~510 nm) for direct excitation of YFP (dYFP), and Yellow light (~575 nm) for excitation of Texas Red (Asano et al. 2024; Tan et al. 2024). A multi‐bandpass filter (cat. no. 69008x; Chroma) was utilized to restrict the excitation light to these designated wavelengths. A multi‐band beamsplitter (cat. no. ZT445/514/594rpc; Chroma) directed the multiple excitation lights to the mice and allowed the emission lights of YFP (both fYFP and dYFP) and Texas Red to pass through for detection. It is important to note that the emission of CFP (fCFP) was not detected in this configuration. The excitation power at the tip of the bare optical fiber inserted into the brain was approximately 3 mW for the Yellow light. The emission lights of YFP and Texas Red were then split with another dichroic mirror (cat. no. FF605‐Di02; Semrock, IL, USA). The wavelengths of the emission lights were restricted using a bandpass filter cat. no. ET539/21x (Chroma) for detecting YFP (fYFP and dYFP) and a bandpass filter cat. no. ET640/30x (Chroma) for detecting Texas Red. The excitation sequence was as follows: the Blue excitation light (~440 nm) was illuminated for 10 ms, followed by a 10 ms interval, then the Teal excitation light (~510 nm) for 10 ms, another 10 ms interval, and finally the Yellow excitation light (~575 nm) for 30 ms. After an interval of 930 ms, the same sequence would commence again. In this configuration, the output waveform within each excitation pulse was averaged, resulting in an actual sampling frequency of 1 Hz for each excitation light.

Using fiber photometry from the hippocampus contralateral to the stimulated hippocampus (Amaral et al. 2007; Wang et al. 2014; Shimoda et al. 2022), we confirmed that the fluorescence fluctuations in response to epileptic neuronal hyperactivity are not caused by unintended artifacts localized to the stimulation electrodes (Figure S1, see Supplementary Discussions).

### 2.5 Data Analysis

No a priori sample size calculation was performed. However, based on our prior experience with inducing epileptic kindling and conducting in vivo fiber photometry in transgenic mice (Ikoma, Sasaki, et al. 2023), the sample size needed for statistical validation was estimated.

Animal seizure behavior was manually evaluated through offline analysis of video recordings. The fluorescence data from photomultiplier tube (PMT) outputs and the electroencephalography (EEG) data were also analyzed offline using AxoGraph (AxoGraph Scientific, Australia). No blinding procedures were implemented in this study. No data were excluded. A Shapiro–Wilk test was conducted to assess whether the data followed a normal distribution. All datasets satisfied the normality assumption and were thus analyzed using parametric statistical methods. Data are presented as means ± standard error of the mean (SEM). Statistical analyses were conducted using Python 3.7.6, employing paired *t*‐tests or Welch's *t*‐tests as appropriate. Statistical significance was set as ***p < 0.001, **p < 0.01, and *p < 0.05. The specific statistical methods used for each individual test are detailed in the figure legends.

## 3 Results

### 3.1 Neuronal ATP Reduced With Epileptic Neuronal Hyperactivity

An optical fiber, along with two tungsten electrodes, was inserted into the mouse hippocampus to deliver a train of electrical stimulation, evoking epileptiform neuronal hyperactivity. This neuronal hyperactivity persisted even after the cessation of the stimulation, leading to the observation of after‐discharges (ADs) recorded via screw electrodes placed on the cortical surface. During the initial rounds of hippocampal stimulation, only the AD was apparent in the recorded cortical EEG, with no observable behavioral seizure responses.

**FIGURE 1:**
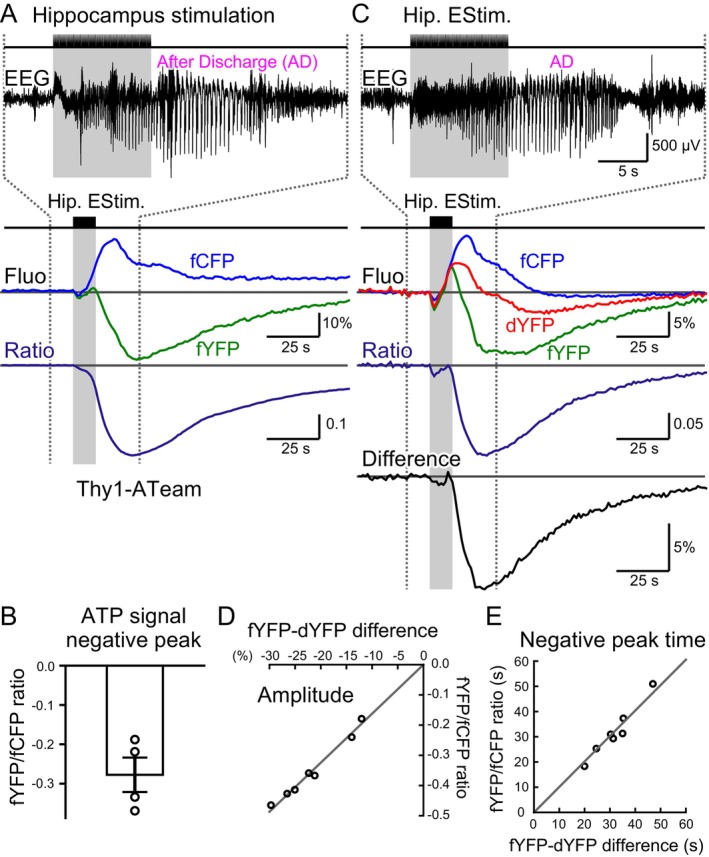
Neuronal cytosolic ATP levels reduced with epileptic neuronal hyperactivity. (A) An optical fiber and a pair of stimulating electrodes were implanted in the hippocampus of a transgenic mouse expressing a FRET‐based fluorescence sensor protein for cytosolic ATP in neurons (Thy1‐ATeam mouse). Screw electrodes were implanted in the skull, with their ends touching the cortical surface for EEG recordings. A train of hippocampal electrical stimulation generated epileptic neuronal hyperactivity, resulting in oscillatory neuronal discharges that persisted even after the cessation of stimulation (after‐discharges; ADs). Excitation light at ~440 nm (Blue) was delivered to the hippocampus via the optical fiber, and both CFP fluorescence (fCFP) and FRET‐mediated YFP fluorescence (fYFP) were measured. With the occurrence of epileptic neuronal hyperactivity, fYFP decreased, while fCFP increased, leading to a decrease in the calculated fYFP/fCFP ratio, suggesting a reduction in neuronal cytosolic ATP concentration. (B) Conventionally, the ratio method (fYFP/fCFP) is widely used in studies employing FRET‐based fluorescence sensor proteins. Using this method, neuronal cytosolic ATP concentration was estimated to decrease with neuronal hyperactivity. Hippocampal stimuli were delivered every hour for 12 sessions during the nighttime. The negative peak values of the ATP signal transients were measured for the first three of the 12 stimulus episodes and averaged. As described later, the series of stimulus episodes induced a kindling effect, where the latter episodes often elicited a stronger seizure response. To focus on the early stage before the kindling effect becomes prominent, only the first three episodes were examined. Data were collected from 4 animals (−0.28 ± 0.04) and are presented as mean ± SEM. (C) The YFP component of the ATeam was directly excited with ~510 nm light (Teal), allowing for measurement of the direct YFP fluorescence signal (dYFP). ATP concentration fluctuations can be estimated using either the ratio method (fYFP/fCFP) or the difference method (fYFP–dYFP). (D) Both the difference method (fYFP–dYFP) and the ratio method (fYFP/fCFP) were used to estimate the peak magnitude of neuronal cytosolic ATP reduction. Each data point represents the average of the first three stimuli of the 12‐stimulus episode series for a single animal (total of 7 animals). The diagonal line represents a linear regression fit to the data, with the XY intercept restricted at the origin (0, 0). (E) The peak times of neuronal cytosolic ATP reduction were also estimated by difference (fYFP–dYFP) and ratio (fYFP/fCFP) method. The scatter plot showed strong correlation, suggesting that either method can be reliably used to evaluate the ATP concentration transients. The diagonal line represents a linear regression fit to the data, with the XY intercept restricted at the origin (0, 0).

Using fiber photometry in transgenic mice expressing the ATP sensor ATeam specifically in the cytosol of neurons (Thy1‐ATeam; Trevisiol et al. 2017), we observed that the CFP fluorescence (fCFP), excited by Blue light (~440 nm) increased, while the YFP fluorescence (fYFP) decreased with hippocampal train stimulation (Figure 1A). By calculating the fYFP/fCFP ratio, we estimated a decrease in neuronal cytosolic ATP concentration associated with epileptiform activity (Figure 1B).

However, we have previously demonstrated that fluorescence intensity fluctuations recorded using the fiber photometry method can be influenced by changes in local brain blood volume (BBV) and cytosolic pH (Ikoma, Sasaki, et al. 2023; Ikoma, Takahashi, et al. 2023). To further investigate this, we directly measured the fluorescence of YFP (dYFP) excited by Teal light (~510 nm). Increases and decreases in dYFP were observed following hippocampal train stimulation, suggesting fluctuations in BBV and/or neuronal cytosolic pH during epileptiform activity. Assuming that changes in BBV and pH affect fYFP and dYFP equally, the difference between fYFP and dYFP could provide an alternative estimate of cytosolic ATP changes (see Supplementary Discussions for details). The difference method (fYFP–dYFP) yielded qualitatively similar results, indicating a decrease in ATP concentration (Figure 1C). The effects of hippocampal stimulation on neuronal cytosolic ATP signal reduction varied among individual animals. Nonetheless, the ATP signal calculated using either the ratio method or the difference method correlatively decreased following hippocampal stimulation (Figure 1D). The negative peak time of the ATP signal, as estimated using the difference method, also showed nearly perfect correlation with the results obtained using the ratio method (Figure 1E). Throughout the following sections, we used the difference method to analyze cytosolic ATP signal fluctuations (see Supplementary Discussions).

### 3.2 The Magnitude of Neuronal ATP Reduction Does Not Correlate With the AD Duration

**FIGURE 2:**
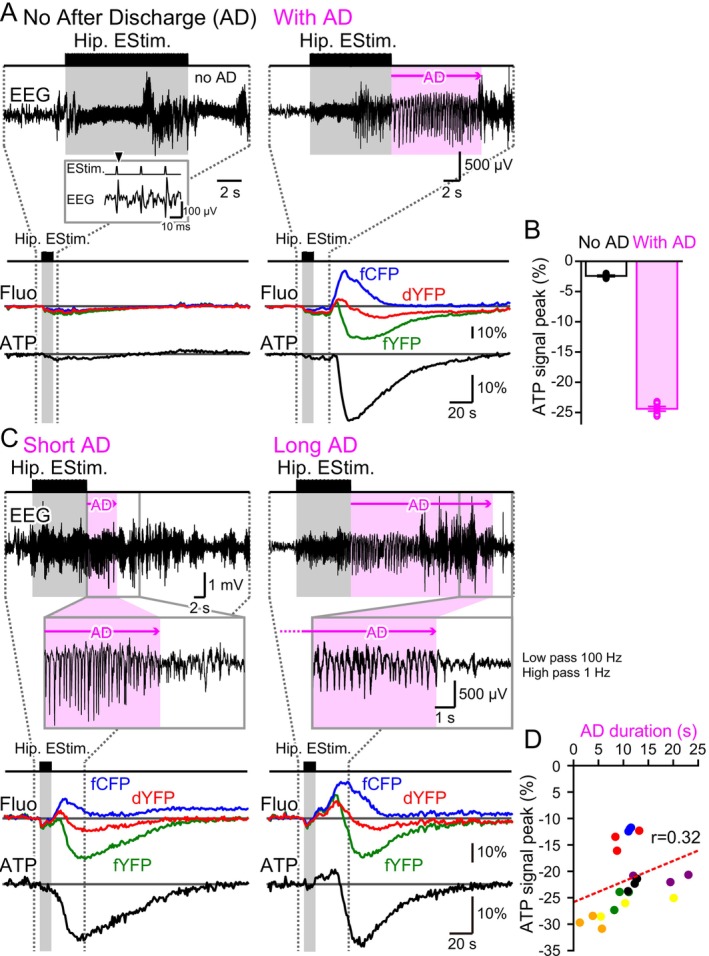
ATP reduction was observed only during AD occurrences, with no correlation between the magnitude of ATP reduction and AD duration. (A) In the same animal, identical hippocampal stimulation sometimes induced AD (With AD, right) and other times did not (No AD, left). When an AD was not generated, the fluorescent signal from the ATeam showed minimal change. The inset shows that even in the ‘No AD’ case, individual electrical stimuli were effective in generating neuronal activity. When an AD was successfully generated, fluctuations in the fCFP, dYFP, and fYFP signals indicated a reduction in neuronal cytosolic ATP levels. Gray shading represents the hippocampal electrical stimulation period, while magenta shading indicates the duration of the AD. (B) The magnitude of ATP reduction with hippocampal electrical stimulation was evaluated using the difference method (fYFP‐dYFP). ATP reduction was significantly more pronounced in the ‘With AD’ condition compared to the ‘No AD’ condition recorded from the same animal (No AD: −2.43 ± 0.13, *n* = 6; With AD: −24.39 ± 0.38, *n* = 6). Similar results were obtained with another mouse; however, in this case, dYFP was not recorded. Thus, the ATP reduction was calculated with the ratio method (fYFP/fCFP) and a significant difference was observed between the conditions (No AD: −0.0068 ± 0.0026, *n* = 3; With AD: −0.1949 ± 0.0087, *n* = 9). (C) AD duration varied even with the same stimulus intensity. The cessation time of epileptic oscillatory neuronal discharges was determined through manual examination of EEG traces. The frequency band was constrained to 1–100 Hz by digital filtering and viewed at high magnification for clearer manual determination of AD cessation time, as shown in the insets. This filter effectively removed contamination from EMG signals and motion artifacts, enabling the examination of typical epileptic slow oscillations. Neuronal cytosolic ATP reduction magnitude was consistent regardless of AD duration (left: Short AD; right: Long AD). (D) The magnitude of ATP reduction was plotted against AD duration. The ATP reduction in response to the first three episodes of hippocampal stimulation in a series was examined and plotted. The red dashed line represents the calculated correlation (r = 0.32). Data from the same animal are shown in the same color, with data from 7 animals presented.

Especially during the early trials of hippocampal electrical stimulation, the same intensity of stimulation occasionally produced epileptic ADs, while at other times it failed to do so. As shown in the inset of Figure 2A, although individual stimulation pulses effectively stimulated neurons, the train stimulation did not always initiate autonomous oscillatory neuronal activity. In these cases, negligible changes were observed in all fluorescence traces (fYFP, fCFP, dYFP) (Figure 2A left). In contrast, when the stimulation successfully induced oscillatory neuronal activity, a typical reduction in ATP signal levels was observed (Figure 2A right). In this particular mouse, ADs were generated in 6 out of 12 trials. Notably, ATP signal levels prominently decreased only in trials where a successful AD occurred (Figure 2B).

In trials with successful ADs, the duration of the AD varied significantly between trials. The insets of Figure 2C show close‐ups of the timing of AD cessation. Regardless of whether the AD was short or long, ATP signal levels decreased by a similar amplitude (Figure 2C). When the amplitude of ATP signal decrease was plotted against AD duration, no negative correlation was found (Figure 2D).

### 3.3 BBV and ATP Changes Associated With Epileptic Neuronal Hyperactivity

The dYFP signal from ATeam is not expected to change with fluctuations in cytosolic ATP. However, in most trials, an increase in dYFP fluorescence was observed approximately 0 to 20 s after the cessation of hippocampal train stimulation and the occurrence of epileptic after‐discharges (ADs), followed by a decrease. Since the fluorescent ATeam is expressed in neurons within the brain parenchyma and blood vessels exhibit minimal autofluorescence at the wavelength used, blood vessels appear as dark shadows in fluorescence imaging (Sasaki et al. 2024). Fluctuations in vessel diameter and local BBV would lead to mirrored changes in dYFP fluorescence intensity detected through fiber photometry. Thus, an increase in the dYFP signal may indicate blood vessel constriction and a decrease in BBV.

**FIGURE 3:**
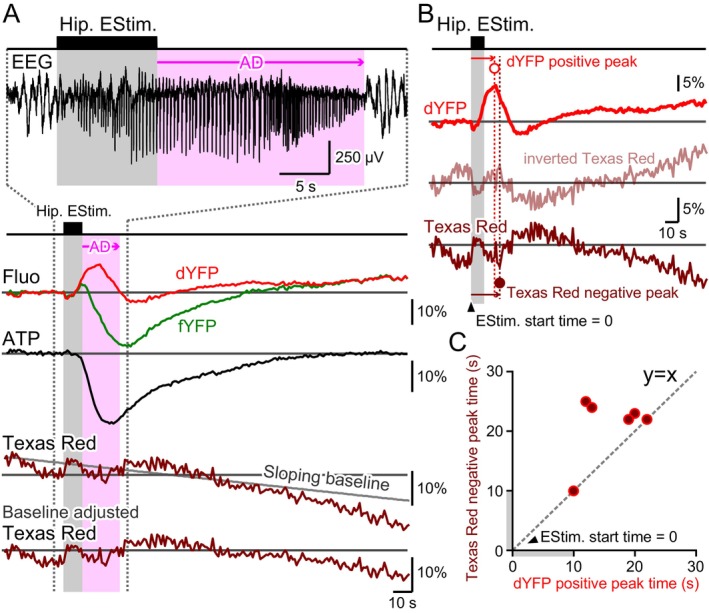
Local brain blood volume (BBV) fluctuations associated with epileptic neuronal hyperactivity. (A) A train of hippocampal electrical stimulation elicited an after‐discharge (AD) response (EEG; the shown trace was band‐pass filtered at 1–100 Hz), resulting in fluctuations in fYFP (green) and dYFP (red) signals. A prominent reduction in neuronal cytosolic ATP was estimated using the difference method (fYFP–dYFP). Although dYFP from the ATeam fluorescence signal is insensitive to ATP concentration fluctuations, a notable increase in dYFP was observed during the occurrence of AD. This increase may be attributed to either local BBV reduction or neuronal cytosolic pH alkalinization. To directly evaluate BBV fluctuations, Texas Red was injected into the tail vein to visualize blood vessels (brown). As the intensity of the Texas Red fluorescence signal rapidly decayed post‐injection, a sloping baseline was fitted to adjust the Texas Red signal. The Texas Red signal decreased during the AD period, suggesting constriction of blood vessels and a subsequent reduction in local BBV adjacent to the tip of the optical fiber. (B) The timing of the positive peak of the dYFP signal (open red circle) was compared with the timing of the negative peak of the Texas Red signal (solid brown circle), both occurring at similar times. The Texas Red signal (brown trace) was inverted and represented in the middle row trace (semi‐transparent brown trace). The shape of the inverted Texas Red trace resembled that of the dYFP trace (red trace); however, it is important to note that the positive peak in the dYFP signal was much more pronounced than that in the inverted Texas Red trace. Therefore, the dYFP signal may also be influenced by pH changes in the neuronal cytosol. (C) The timing of the negative peak of the Texas Red signal was plotted against the positive peak of the dYFP signal, with a diagonal gray dashed line (*y* = *x*) drawn for reference. Only the first epileptic episode induced by hippocampal stimulation following the tail vein injection of Texas Red was analyzed, as the Texas Red signal was clearly detectable only during the first hour after injection. Therefore, each data point presents an individual measurement from one animal (*n* = 6 mice). The positive peak in dYFP occurred either closely in time with, or preceded, the negative peak in Texas Red by as much as approximately 10 s. These data suggest that, while fluctuations in the dYFP signal primarily reflect the shadow effect from BBV changes, other factors, such as cytosolic pH, may also play a role.

To evaluate whether BBV fluctuates with epileptic neuronal hyperactivity induced by hippocampal electrical stimulation, Texas Red dextran, a fluorescent dye, was injected into the tail vein. The fluorescence of the dye in blood vessels was measured alongside the fYFP and dYFP signals emitted from neuronal ATeam using the optical fiber implanted in the hippocampus. Texas Red rapidly circulates throughout the body, and its fluorescence can be observed in vivo in brain tissue immediately after injection. However, due to the dilution of Texas Red, its fluorescence typically decays rapidly over a time course of 1 to 2 h, creating a limited window for direct blood volume measurements. The detected Texas Red fluorescence generally resides on a sloping baseline, so a linear fit of the baseline trend was subtracted from the recorded trace (Figure 3A). Typically, a corresponding increase in dYFP intensity upon the occurrence of epileptic ADs was accompanied by a decrease in Texas Red fluorescence (Figure 3B). The inverted Texas Red trace closely resembled the dYFP trace, indicating that changes in BBV influence the dYFP signals. However, the positive peak in the dYFP trace, which coincides with the occurrence of epileptic AD, was often more pronounced than the negative peak in Texas Red. This suggests that factors other than BBV fluctuations may also affect the dYFP traces.

The timing of the negative peak in the Texas Red signal was closely compared to the positive peak in the dYFP trace (Figure 3C). The negative peak in Texas Red occurred either closely in time with, or lagged behind, the positive peak in dYFP by as much as approximately 10 s. This may also suggest that fluctuations in dYFP do not completely reflect inverted BBV changes, and that other factors, such as cytosolic pH, may play a role.

**FIGURE 4:**
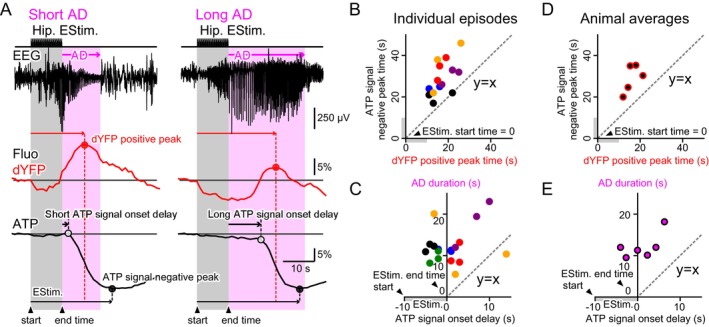
ATP signal reduction onset occurs prior to after‐discharge (AD) cessation. (A) Hippocampal electrical stimulation sometimes induced a short AD duration, while at other times, it resulted in a longer AD (EEG; the shown trace was band‐pass filtered at 1–100 Hz). The positive peak in the dYFP signal (red circle) occurred earlier than the negative peak in neuronal cytosolic ATP signal reduction (black circle). The onset of the ATP signal reduction was determined by identifying the negative peak of the second derivative of the ATP signal trace (i.e., the inflection point). (B) The timing of the negative peak in the ATP signal was plotted against the timing of the positive peak in the dYFP signal. Data were derived from the first three episodes (out of 12) delivered during the nighttime of the first day of kindling. Data points from the same animal are shown in the same color, with data from 5 animals included. In one case, the recorded dYFP trace did not exhibit a prominent positive peak, so the data from this animal were excluded. The original fluorescence traces from this particular mouse are shown in Figure S3. The diagonal gray dashed line represents a proportional line with a ratio of 1 (*y* = *x*). (C) The duration of the AD was plotted against the delay in the onset of ATP signal reduction. Data from the same animal are shown in the same color, with data from 6 animals presented. The AD duration and ATP signal onset delay could be measured in the recordings from the mouse excluded in panel B (Figure S3); therefore, the total number of animals included is 6. The diagonal gray dashed line represents a proportional line with a ratio of 1 (*y* = *x*). (D) Summary data of the ATP signal negative peak time plotted against the dYFP signal positive peak time from 5 animals. Each data point represents the average of the first three hippocampal stimulus episodes from each animal. All data points fell above the dotted diagonal line, indicating that the negative peak of the ATP signal consistently occurred later than the positive peak of the dYFP signal. (E) Summary data of AD duration plotted against the delay in the onset of ATP signal reduction from 6 animals. Each data point represents the average of the first three hippocampal stimulus episodes from each animal.

We established that the positive shift in dYFP following epileptic ADs partially corresponds to a decrease in local BBV. The timing of the dYFP positive peak was compared with the negative peak of the estimated ATP change (Figure 4A). It was observed that the longer it took for dYFP to reach its positive peak, the longer it took for ATP to decrease to its negative peak. Notably, the ATP negative peak consistently lagged behind the dYFP positive peak (Figure 4B,D). These observations suggest that a decrease in blood flow and net ATP concentration occurs concurrently.

### 3.4 Pyruvate Increase in Astrocytes

**FIGURE 5:**
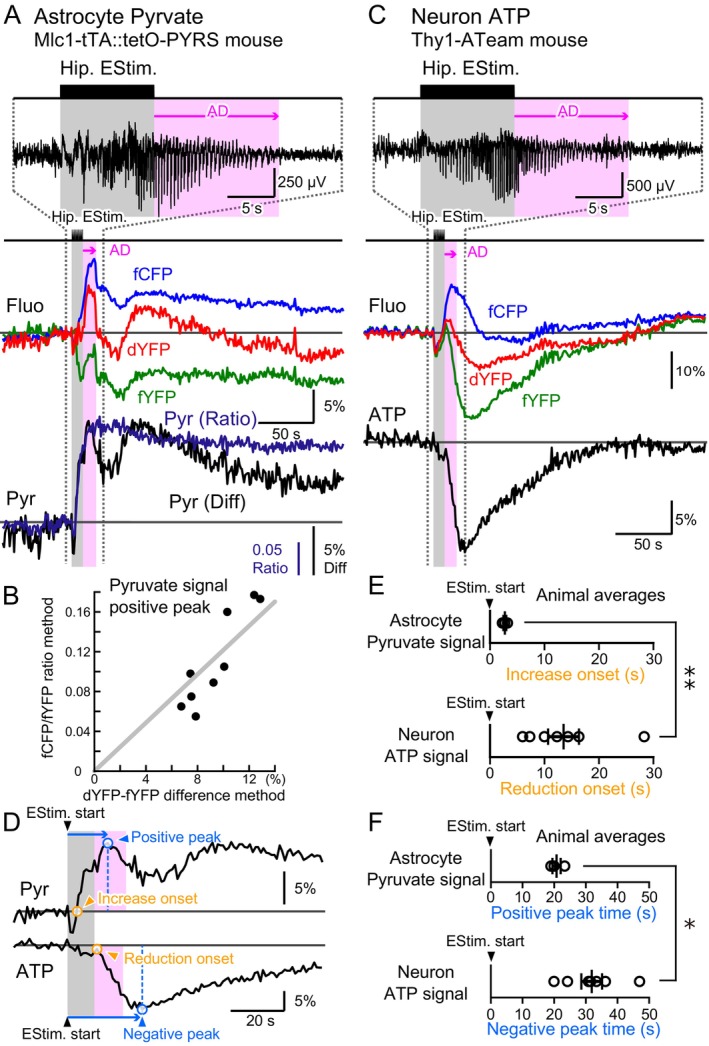
Rapid increase in pyruvate signal in astrocytes. (A) A FRET‐based cytosolic pyruvate fluorescence sensor was specifically expressed in astrocytes (Mlc1‐tTA::TetO‐PYRS transgenic mice). An optical fiber and a pair of stimulating electrodes were implanted into the hippocampus. Following a train of hippocampal stimulation, epileptic AD was generated (EEG; the shown trace was band‐pass filtered at 1–100 Hz), and fluctuations in the fYFP (green), fCFP (blue), and dYFP (red) signals were observed. In contrast to the binding of ATP to ATeam, FRET efficiency is expected to decrease upon the binding of pyruvate to PYRS. Astrocytic pyruvate concentration transients were estimated using either the ratio method (fCFP/fYFP) or the difference method (dYFP–fYFP). Tri‐phasic pyruvate concentration dynamics was estimated using the difference method. The onset of the first phase of the pyruvate signal increase was defined as the point where the signal exceeds the baseline. The positive peak of the pyruvate signal was also identified. (B) The positive peak of the pyruvate signal estimated using the ratio method was plotted against that obtained with the difference method (*n* = 9 episodes from 3 animals). A linear regression line was fitted to the data plot with a fixed *y*‐intercept at (0, 0). (C) A representative recording from a Thy1‐ATeam mouse. (D) Representative recordings of the astrocytic PYRS signal and the neuronal ATeam signal were vertically aligned on a close‐up time scale. The astrocytic pyruvate signal exhibited a transient negative deflection followed by a steep increase. The onset of the pyruvate signal increase was defined as the point where the pyruvate signal crossed the baseline. The neuronal ATP signal decreased following hippocampal stimulation. The onset of the ATP signal reduction was determined by identifying the negative peak of the second derivative of the ATP signal trace (i.e., the inflection point). Additionally, the positive peak of the pyruvate signal and the negative peak of the ATP signal were identified. (E) The onset of the first phase of the pyruvate signal increase in astrocytes (top, 2.72 ± 0.27, *n* = 3 animals) and the onset of the ATP signal reduction in neurons (bottom, 13.50 ± 2.83, *n* = 7 animals) were compared. A significant difference in the onset times was found between the astrocytic pyruvate signal and the neuronal ATP signal (Welch's *t*‐test, degree of freedom = 8, *t* = −3.789, *p* = 0.008, data presented as the mean ± SEM). (F) The initial positive peak of the pyruvate signal corresponds to the onset of the signal's decrease from its maximum (top, 20.75 ± 1.31 s, *n* = 3 animals). This was compared with the time of the negative peak of the ATP signal in neurons (bottom, 31.86 ± 3.28 s, *n* = 7 animals). A significant difference was found between these times (Welch's *t*‐test, degree of freedom = 8, *t* = −3.148, *p* = 0.015, data presented as the mean ± SEM). In addition to individual data points, the mean ± SEM is presented. Statistical significance was set as *p < 0.05 and **p < 0.01.

To investigate energy dynamics between astrocytes and neurons during epileptic neuronal hyperactivity in the hippocampus, we focused on pyruvate, a key intermediate in glucose metabolism. We generated a transgenic mouse line (Mlc1‐tTA::tetO‐PYRS) that expresses a FRET‐based sensor for pyruvate (PYRS) (Niino et al. manuscript in preparation) specifically in astrocytes. Notably, unlike ATeam (Figure 5C), the FRET efficiency of PYRS is expected to decrease upon ligand binding.

During epileptic ADs induced by electrical stimulation, the fYFP signal of PYRS in astrocytes decreased while the fCFP signal increased, indicating an increase in cytosolic pyruvate levels in astrocytes (Figure 5A). The dYFP signal of PYRS also exhibited prominent fluctuations, suggesting local BBV changes similar to those observed with ATeam. As with ATeam, the ratio method (fCFP/fYFP) and the difference method (dYFP–fYFP) produced highly correlated results, accurately capturing pyruvate signal dynamics (Figure 5B). The following analysis is based on pyruvate signals calculated using the difference method.

The increase in the pyruvate signal typically followed a triphasic time course (Figure 5A). Initially, a rapid positive transient (1st phase) was observed. The 2nd phase was characterized by either (a) a slow increase, peaking approximately 1 min after the onset of electrical stimulation and lasting for a couple of minutes, or (b) a slow decay, settling into a new steady state within a few minutes. In both cases, the signal eventually reached a sustained steady state (3rd phase), significantly higher than the pre‐stimulation baseline and persisting for up to ~40 min.

Notably, the increase in astrocytic cytosolic pyruvate signal preceded the reduction in neuronal ATP signal (Figures 5D,E and S2A). Finally, we compared the timing of the astrocytic pyruvate signal's positive peak with the neuronal cytosolic ATP signal's negative peak (Figures 5D,F and S2B). After reaching its negative peak, neuronal ATP signal levels began to recover, following the decline of the astrocytic pyruvate signal.

### 3.5 Neuronal ATP Decreases Less With Exacerbation of Epilepsy

**FIGURE 6:**
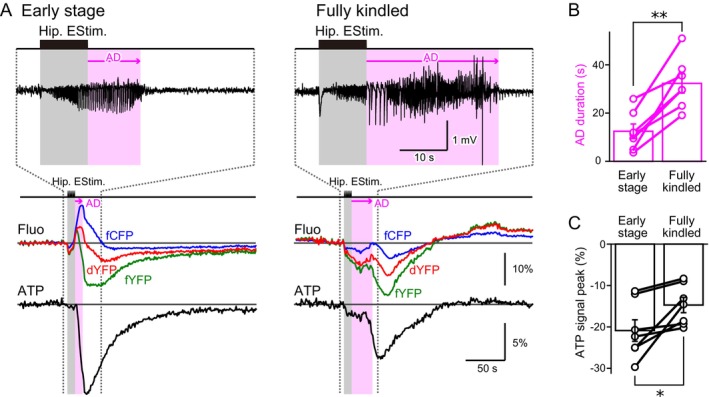
The magnitude of neuronal cytosolic ATP signal reduction decreases with the exacerbation of epileptic seizures by kindling. (A) With repeated hippocampal stimulation, the exacerbation of epileptic seizures occurs through a process called kindling. After‐discharge (AD) duration was short‐lasting in the Early stage (left) and became prolonged in the Fully kindled stage (right) (EEG; the shown trace was band‐pass filtered at 1–100 Hz). Near‐mirrored images of fCFP and fYFP were observed with small changes in the dYFP during the Early stage, while the three fluorescence traces followed a more complex time course in the Fully kindled stage. The magnitude of ATP signal reduction was estimated using the difference method (fYFP–dYFP). Neuronal cytosolic ATP signal reduction in response to epileptic hippocampal electrical stimulation became less pronounced in the Fully kindled stage. (B) AD duration was significantly prolonged in the Fully kindled stage compared to the Early stage (Early stage 12.46 ± 3.03 s, Fully kindled 32.31 ± 3.99 s, *n* = 7 animals, paired *t*‐test, degree of freedom = 6, *t* = −5.75 *p* = 0.001). Each data point represents the mean of the AD durations in response to the first three or the last three epileptic episodes of kindling for the Early stage and Fully kindled, respectively. In addition to individual data points, the mean ± SEM is shown. (C) The magnitude of ATP signal reduction was less in the Fully kindled stage compared to the Early stage (Early stage −20.88% ± 2.60%, Fully kindled −14.72% ± 1.84%, *n* = 7 animals, paired *t*‐test, degree of freedom = 6, *t* = −3.04, *p* = 0.023). Each data point represents the mean of the first three or the last three epileptic episodes of kindling for the Early stage and Fully kindled, respectively. In addition to individual data points, the mean ± SEM is shown. Statistical significance was set as *p < 0.05 and **p < 0.01.

Kindling is a process in which epileptic neuronal hyperactivity gradually intensifies with repeated electrical stimulation of the hippocampus. In this study, we administered hippocampal electrical stimulation 12 times per night over several consecutive days to induce kindling. As kindling progressed, we observed a notable increase in the duration of epileptic ADs, accompanied by an exacerbation of behavioral seizure activity. On the first day of kindling, animals typically exhibited no discernible behavioral responses, classified as Stage A (Figure 6A, left; Ikoma, Sasaki, et al. 2023). Despite this lack of visible behavioral changes, short‐lasting epileptic ADs were already present. However, as kindling advanced, animals began displaying more pronounced reactions, such as tail twitching and lying down, categorized as Stage B. In the final stages of kindling (Stage C), wild running behaviors emerged, along with prolonged epileptic AD episodes (Figure 6A, right). A fully kindled state was defined as either the occurrence of at least five Stage C responses within 12 stimulation episodes or five consecutive Stage C responses, including episodes from the previous day. This state was typically reached within 3 to 5 days of stimulation.

Since fully kindled mice exhibited significantly prolonged ADs (Figure 6B), one might expect greater ATP consumption in neurons. Consequently, a more pronounced reduction in ATP signal during epileptic neuronal hyperactivity would seem likely in the fully kindled state compared to the early stage. However, our findings revealed the opposite: the neuronal cytosolic ATP signal decreased more in the early stage (Figure 6C; −20.88% ± 2.60% of basal fluorescence, *n* = 7 animals) than in fully kindled mice (−14.72% ± 1.84%). This suggests that the primary factor driving ATP reduction in neurons is not epileptic neuronal hyperactivity itself.

After assessing the fully kindled stage, kindling stimulation was halted, and the experiment was concluded. However, in rare cases, a fully kindled stage was rapidly reached during a nighttime kindling session, resulting in fatal hippocampal stimulation and sudden unexpected death in epilepsy (SUDEP). SUDEP also occurs in human patients, yet its underlying causes and potential preventive measures remain largely unknown, as EEG monitoring is typically unavailable during such incidents (but see, Ryvlin et al. 2013). Coincidentally recorded SUDEP incidents in a mouse model provided a unique opportunity to analyze neuronal cytosolic ATP signal dynamics in detail. Since biochemical processes of cellular respiration cease upon death, cellular ATP concentration is expected to decrease. SUDEP was marked by a substantial drop in neuronal cytosolic ATP signal levels (−38.04% ± 3.37%, *n* = 3 animals, data not shown), confirming the functionality of ATeam as an ATP sensor in vivo (see Supplementary Discussions). In comparison, the mean ATP signal reduction during the early stages of kindling was as much as 55% of that observed in SUDEP, suggesting that epileptic hyperactivity imposes a considerable metabolic challenge to neurons.

### 3.6 Possible Effect of Changes in the Energy Supply

**FIGURE 7:**
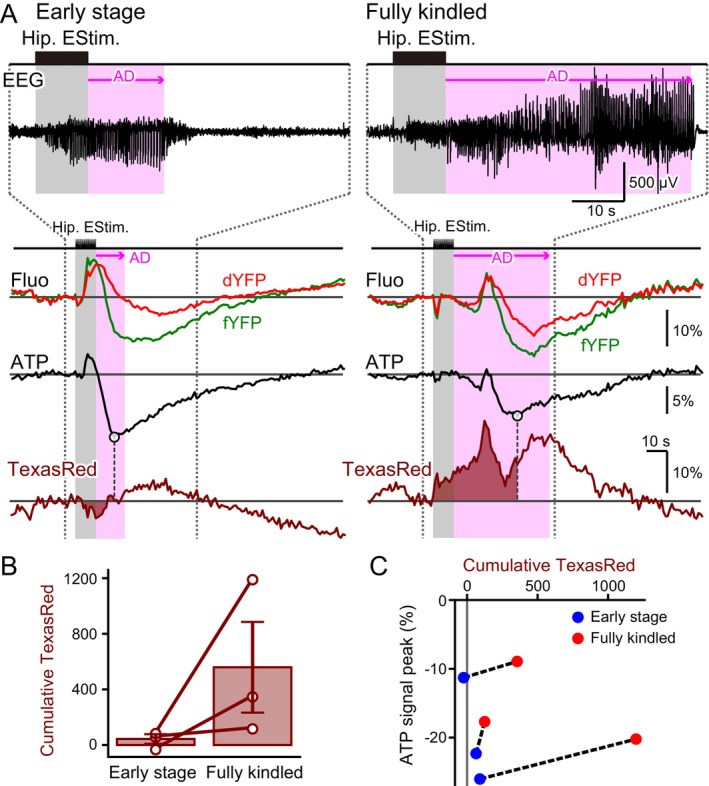
Hippocampal blood flow dynamics change with kindling. (A) A representative trace of fYFP (green), dYFP (red), and Texas Red (brown) responses to epileptic hippocampal electrical stimulation. AD was prolonged in the Fully kindled stage compared to the Early stage (EEG; the shown trace was band‐pass filtered at 1–100 Hz). Neuronal cytosolic ATP signal fluctuations were estimated using the difference method (fYFP–dYFP). The magnitude of ATP signal reduction decreased in the Fully kindled stage. The Texas Red trace reflects local brain blood volume dynamics. The dYFP signal is affected by both the shadow effect of the BBV and fluctuations in cytosolic pH. While the Texas Red and dYFP traces were nearly mirrored, the dYFP deviated from the inverted Texas Red trace at certain points, likely where large fluctuations in cytosolic pH occurred. The white circles indicate the timing of the ATP signal negative peak. The brown shading represents the cumulative Texas Red signal from the start of electrical stimulation to the ATP signal negative peak. (B) Cumulative Texas Red fluorescence was consistently positive and larger in the Fully kindled stage compared to the Early stage; however, this difference was not statistically significant (Early stage 43.37 ± 34.84 a.u., Fully kindled 559.28 ± 326.24 a.u., *n* = 3 animals, paired *t*‐test, degree of freedom = 2, *t* = −1.666, *p* = 0.238). In addition to individual data points, the mean ± SEM is shown. (C) The relationship between ATP signal reduction and cumulative Texas Red fluorescence was plotted. Cumulative Texas Red always increased with kindling, but the magnitude of ATP signal reduction decreased from the Early stage (blue dot) to the Fully kindled stage (red dot) within the data from the same animal (data pairs from the same animal are connected by straight dashed lines).

During the early stage of kindling, an increase in dYFP was observed upon the generation of epileptic ADs, potentially indicating a decrease in blood flow. A single exception (Figure S3) showed no apparent positive deflection in dYFP fluorescence during this stage. As kindling progressed, the duration of ADs progressively increased (Figures 6 and 7). Correspondingly, the dYFP increase became less pronounced, and its waveform grew more complex, often followed by a subsequent decrease in dYFP fluorescence (Figures 6 and 7).

To directly assess local blood volume changes, Texas Red was injected just before the first hippocampal electrical stimulation on Day 1. Since Texas Red fluorescence fades over time, it is no longer detectable in subsequent kindling sessions. After the mouse reached the fully kindled stage, Texas Red was reinjected to visualize blood volume dynamics. Simultaneous recordings of fYFP and dYFP changes were conducted to evaluate alterations in neuronal cytosolic ATP concentration.

Compared to the early stage of kindling, a prominent increase in Texas Red fluorescence was observed at the fully kindled stage following hippocampal electrical stimulation (Figure 7A). ATP fluctuations were estimated using the difference method, and the negative peak was measured. The cumulative change in Texas Red fluorescence relative to the baseline was calculated up to the time point of this ATP signal negative peak. In all three recorded animals, the cumulative Texas Red signal was higher at the fully kindled stage than in the early stage, although the difference was not statistically significant (Figure 7B). Additionally, cumulative Texas Red signal changes were calculated up to the end of ADs (Figure S4). While a trend toward increased blood volume with kindling was observed, no statistically significant difference was detected. As shown in Figure 6, ATP signal reduction was less pronounced in the fully kindled stage compared to the early stage (Figure 7C). These findings suggest that as epileptic neuronal hyperactivity progresses, blood supply increases, which in turn mitigates the reduction in neuronal cytosolic ATP.

## 4 Discussion

We observed a prominent decrease in neuronal cytosolic ATP levels in response to epileptic neuronal hyperactivity. Cytosolic ATP concentration is determined by the balance between its consumption and supply. This net decrease could result from one or more of the following: (A) an increase in ATP consumption within neurons, (B) a decrease in the supply of energy‐generating substrates to neurons, and/or (C) a decrease in ATP production within neurons. We first considered the possibility of (A) increased ATP consumption.

Neurons are electrically dynamic cells; during action potential firing and synaptic activity, extracellular Na^+^ flows into neurons, while intracellular K^+^ flows out. It is generally assumed that neurons expend most of their energy to power Na^+^/K^+^‐ATPase, which helps restore and maintain the ionic imbalance across the cell membrane. Studies have also shown that the brain's metabolic energy supply system likely accommodates a wide range of neuronal electrical activity frequencies, allowing neuronal cytosolic ATP concentrations to remain stable (Baeza‐Lehnert et al. 2019). However, this ATP stability breaks down once epileptic ADs are generated (Figure 2A). Intuitively, one might expect a larger decrease in ATP levels with longer AD durations. However, our data show that increased epileptic neuronal discharges do not lead to a corresponding increase in the reduction of neuronal cytosolic ATP levels (Figure 2C,D). In some episodes, nearly 10 s of repetitive neuronal electrical activity occurred before a drastic reduction in ATP levels was observed (Figure 4). This suggests that ATP may not be predominantly used to restore ionic imbalance during repetitive action potential firing in epileptic neuronal hyperactivity. The substantial decrease in neuronal cytosolic ATP during epileptic neuronal hyperactivity could be driven by a significant increase in ATP consumption specifically linked to AD generation, rather than by the direct restoration of ionic imbalance caused by repetitive action potential firing itself. During epileptic neuronal hyperactivity, a large influx of Na^+^ may occur through mechanisms independent of action potential firing or direct postsynaptic electrical activity, such as through the Na^+^‐bicarbonate cotransporter (NBC) or transient receptor potential vanilloid 4 (TRPV4) channels. ATP may be largely consumed by Na^+^/K^+^‐ATPase to restore the ionic imbalance disrupted by this abnormal Na^+^ influx (Park et al. 2010; Everaerts et al. 2023; Pape and Rose 2023).

Unexpectedly, the reduction in ATP signal was more pronounced in the early stages of epileptic kindling than in the fully kindled state, despite the longer duration and greater complexity of neuronal action potential firing in the fully kindled state. A higher level of plasticity may occur during the early stages of epileptic kindling. Such plasticity could become saturated in the fully kindled state, with further changes in the hyperactive neuronal circuitry no longer progressing. Excess ATP consumption in the early stages of kindling might support this heightened plasticity. If this is the case, neuronal cytosolic ATP levels could be more influenced by the degree of plasticity than by the frequency of neuronal action potential firing. Approximately 25% of total ATP consumption in the brain is estimated to support lipid synthesis, protein synthesis, and cytoskeletal rearrangements (Harris et al. 2012). Neuronal cytosolic ATP might be allocated to these processes, enabling long‐term adjustments in neuronal circuitry during kindling. Actual plastic changes in neuronal circuitry may take minutes to days to manifest; however, the mechanisms that trigger the cascade of events leading to plasticity likely require metabolic energy and occur within seconds. It would also be interesting to examine whether neuronal cytosolic ATP can fluctuate in response to the high energy demands of physiological learning and memory processes (Bohmbach et al. 2023; Fernández‐Moncada et al. 2024). A study has shown that simple whisker stimulation or electrical stimulation of the cortex in vivo, even at frequencies up to 100 Hz, did not produce a noticeable change in the neuronal cytosolic ATP signals (Baeza‐Lehnert et al. 2019). In contrast, a physiological transition to REM sleep resulted in a reduction in the neuronal cytosolic ATP signal, despite increased cerebellar blood flow and presumably higher supplies of energy‐generating substances such as glucose and oxygen (Natsubori et al. 2020). This suggests that a physiological increase in ATP consumption may indeed produce a detectable decrease in ATP signals.

A prominent decrease in neuronal cytosolic ATP levels may result from (B) a decrease in the supply of energy‐generating substrates to neurons. Under basal conditions, cytosolic ATP levels may remain stable due to a dynamic equilibrium between the rapid rates of both supply and consumption. If this balance is disturbed, even a slight hindrance in the supply could lead to a drastic reduction in net ATP levels. The ultimate source of these substances is the blood vessels in the brain. As neurons do not have direct contact with blood vessels, astrocytes could play a crucial role in ensuring an optimal energy supply to neurons. Upon neuronal hyperactivity, we observed a rapid increase in the cytosolic pyruvate signal in astrocytes, followed by a reduction in the cytosolic ATP signal in neurons. Astrocytes can release lactate through monocarboxylate transporters (MCTs), and it has been shown that lactate release can also occur through ion channels (Sotelo‐Hitschfeld et al. 2015; Beppu et al. 2021; Kanaya et al. 2023). When the efficiency of either of these pathways is reduced, an increase in pyruvate and lactate concentrations in astrocytes is expected. In cultured cells, it has been reported that, in the presence of glucose, inhibition of pyruvate and lactate efflux by applying an MCT blocker led to a progressive accumulation of intracellular pyruvate (San Martín et al. 2014). If ATP production in neurons largely depends on the supply of pyruvate/lactate from astrocytes, the transient suppression of pyruvate/lactate release from astrocytes upon epileptic neuronal hyperactivity could lead to a decrease in cytosolic ATP levels in neurons.

After the initial increase in the astrocytic pyruvate signal, we observed a slow reduction, which may reflect a gradual recovery of MCT efficiency from its suppression. The astrocytic pyruvate signal stabilized within a couple of minutes at a level still above the initial baseline prior to stimulation. The elevated cytosolic pyruvate then slowly returned to the initial baseline over a ~40‐min time course. Other mechanisms, beyond the reduction in MCT efficiency, may contribute to maintaining the elevated cytosolic pyruvate concentration above the initial baseline for such an extended period. For instance, a reduction in the rate of mitochondrial metabolism (Lerchundi et al. 2015) could decrease the consumption of cytosolic pyruvate by astrocytic mitochondria. In addition to (1) a reduction in pyruvate/lactate efflux or (2) changes in pyruvate consumption by mitochondria, astrocytic cytosolic pyruvate may increase due to (3) acceleration of glycolysis and enhanced pyruvate production in astrocytes. Excess extracellular K^+^ has been shown to depolarize astrocytes and activate NBCs, causing influx of HCO_3_^−^ (Choi et al. 2012) and resulting in intracellular alkalization (Onodera et al. 2021). In the current study, we also observed indications of astrocytic alkalization, evidenced by an increase in the dYFP signals upon epileptic neuronal hyperactivity (Figure 5A, see Supplementary Discussions). Cytosolic alkalization would result in a decrease in the pH gradient between the cytosol and the alkaline mitochondria and cause reduced uptake of pyruvate into the mitochondria (Lerchundi et al. 2015). Increased intracellular HCO_3_^−^ has also been shown to elevate cAMP, facilitating glycolysis and the production of pyruvate/lactate (Choi et al. 2012). It seems reasonable to assume that excess cytosolic pyruvate, created either by (2) a deceleration of mitochondrial metabolic processes or (3) the acceleration of glycolysis activated by HCO_3_^−^ influx in astrocytes, becomes available for transfer to neurons, aiding in their rapid energy recovery.

Taking these possibilities into consideration, the initial rapid increase in astrocytic cytosolic pyruvate could be attributed to (1) a reduction in pyruvate/lactate efflux, as this increase coincides with and precedes the decrease in neuronal cytosolic ATP levels. The subsequent recovery from ATP depletion in neurons may be supported by increased pyruvate/lactate production in astrocytes through (2) mitochondrial metabolic deceleration and/or (3) glycolysis acceleration, along with a relaxation of (1) pyruvate/lactate efflux suppression in astrocytes.

It has been reported that MCT1 expression is increased in hippocampal astrocytes in both human epileptic patients and in animals with experimentally induced temporal lobe epilepsy (Lauritzen et al. 2012). Therefore, an efficient astrocyte‐mediated energy supply may occur in the hippocampus of the epileptic brain. This increase in supply may help explain the decrease in the magnitude of neuronal cytosolic ATP reduction with the progression of kindling.

Blood vessel constriction in response to epileptic neuronal hyperactivity during the early stages of kindling may be responsible for (B) the decrease in the supply of energy‐generating substrates to neurons. As blood vessels are the ultimate source of energy‐generating substances in the brain, their constriction or dilation can adjust the local supply of these substances to neurons. We observed changes in local brain blood volume (BBV) dynamics in response to epileptic neuronal hyperactivity. A transient decrease in hippocampal local blood flow was noted in the early stages of kindling, whereas an increase in blood flow was observed in the fully kindled stage. It is possible that the significant reduction in neuronal cytosolic ATP levels during the early stages of epileptic neuronal hyperactivity is partially attributable to decreased glucose and oxygen supply resulting from blood vessel constriction. The alleviation of ATP reduction in the fully kindled stage may be due to an enhanced supply of energy‐generating substrates by blood vessel dilation. The amount of energy supplied to neurons may be one of the crucial factors determining the net concentration of neuronal cytosolic ATP. The mechanisms underlying the plastic changes in blood vessel dynamics within the epileptic brain remain to be further explored (Ogaki et al. 2020; Ozawa et al. 2023; Sasaki et al. 2024).

Fluctuations in (C) ATP generating mechanisms within neurons may play a key role in determining the final ATP concentration in the neuronal cytosol. The efficacy of mitochondrial function in neurons could serve as the rate‐limiting factor for ATP production. As suggested in our study, neuronal cytosolic pH appears to transiently increase in response to epileptic neuronal hyperactivity during the early stages of kindling (see Supplementary Discussions). This pH shift may reduce the pH gradient between the cytosol and mitochondria, potentially inhibiting pyruvate uptake into the mitochondria (Lerchundi et al. 2015). Mitochondrial function may temporarily shut down during epileptic neuronal hyperactivity, leading to a drastic reduction in neuronal cytosolic ATP levels. With kindling, neuronal metabolism may adapt to the heightened demands caused by repeated epileptic neuronal hyperactivity, allowing for increased ATP production per unit time, which may limit the reduction in neuronal cytosolic ATP levels. It is also worth noting that, with continued epileptic kindling, the transient increase in dYFP in neurons gradually attenuates (Figure 6), potentially reflecting changes in the metabolic state of neurons over the course of kindling.

Studies also indicate that when neurons are stimulated, they rely more on their glucose metabolism than on lactate derived from astrocytes for energy production (Díaz‐García et al. 2017). In the resting state, the brain's glucose‐to‐oxygen consumption ratio is close to a stoichiometric 1:6, suggesting that nearly all the glucose supplied to the brain is oxidized by mitochondrial function. Upon stimulation, cerebral blood flow typically increases along with glucose utilization; however, the increase in oxygen consumption is not as substantial (Fox et al. 1988). The additional glucose may be directly taken up by neurons, allowing rapid ATP production via glycolysis, which does not require oxygen. This transient uncoupling of glycolysis and oxidative phosphorylation in the active brain may serve as a rapid mechanism to meet sudden energy demands. A similar response is observed in fast‐twitch muscle during intense activity. In the early stages of kindling, when epileptic neuronal hyperactivity is induced, neurons may shift toward glycolysis for ATP production to quickly address the increased energy demand. Whether glycolytic ATP production outpaces the more efficient mitochondrial ATP production, however, remains to be elucidated. This shift to less efficient ATP production via glycolysis may underlie the net reduction in ATP levels in neurons during epileptic neuronal hyperactivity.

The large reduction in ATP observed during SUDEP may be attributed to a complete loss of blood flow, leading to an absence of glucose and oxygen supply, similar to ischemia (Lerchundi et al. 2019). Whether neurons are actually “dead” when the EEG flatlines remains uncertain. The lack of fluctuations in extracellularly recorded electrophysiological signals could indicate either a full suppression of neuronal electrical activity due to deep hyperpolarization or a complete depolarization of neurons to a potential matching the extracellular environment. In the latter case, a significant Ca^2+^ influx would be expected, with neuronal cytosolic ATP likely consumed in efforts to extrude Ca^2+^ and Na^+^ from the cytosol. Notably, early‐stage hippocampal electrical stimulation produced approximately half of the ATP signal reduction observed during a fatal SUDEP situation. This suggests that initial hippocampal stimulation, even without a noticeable behavioral seizure response, still poses a considerable metabolic challenge to neurons.

This study highlights a potentially critical interaction between astrocytic and neuronal energy metabolism during epileptic activity. The role of astrocytes in modulating epilepsy and neuronal oscillatory discharges through gliotransmitter release or gap junction uncoupling has been well documented (Tian et al. 2005; Oberheim et al. 2008; Ikeda et al. 2020; Nikolic et al. 2019; Wang et al. 2020; Gobbo et al. 2021; Shigetomi et al. 2024). Our data suggest that the mechanisms governing energy supply in blood vessels and astrocytes may play a pivotal role in determining the final net ATP concentration levels in neurons during epilepsy. Additionally, exploring whether the level of readily available ATP influences the likelihood of plastic changes in neuronal circuits would be valuable. If this is the case, the metabolic‐neuronal interaction could contribute to the regulation of metaplasticity in the brain as well as to the exacerbation of epilepsy.

## 5 Author Contributions

**Kota Furukawa:** conceptualization, investigation, data curation, formal analysis, visualization, writing – original draft, writing – review and editing, software. **Yoko Ikoma:** methodology, supervision, funding acquisition, writing – review and editing. **Yusuke Niino:** methodology, writing – review and editing, resources. **Yuichi Hiraoka:** methodology, writing – review and editing, resources. **Kohichi Tanaka:** methodology, writing – review and editing, resources, funding acquisition. **Atsushi Miyawaki:** resources, methodology, writing – review and editing. **Johannes Hirrlinger:** methodology, resources, writing – review and editing. **Ko Matsui:** writing – original draft, conceptualization, supervision, funding acquisition, project administration, visualization, writing – review and editing, validation, investigation.

## 7 Ethics Statement

All animal procedures involved in this study were approved in advance and conducted in accordance with the Regulations for Animal Experiments and Related Activities at Tohoku University (2019LsA‐017).

## 8 Conflicts of Interest

The authors declare no conflicts of interest.

9

### 9.2 Peer Review

The peer review history for this article is available at https://www.webofscience.com/api/gateway/wos/peer‐review/10.1111/jnc.70044.

## Supporting information


**Data S1.** Supporting Information.

## Data Availability

The data supporting the findings of this study are available from the corresponding author on reasonable request.
