# Supplementary material for: Dynamics of Neuronal and Astrocytic Energy Molecules in Epilepsy
Source: J Neurochem. 2025 Mar 20;169(3):e70044. doi: 10.1111/jnc.70044 (PMC11923518; doi:10.1111/jnc.70044)

Journal of Neurochemistry

**Supplementary Information**

Dynamics of neuronal and astrocytic energy molecules in epilepsy

Kota Furukawa^1^, Yoko Ikoma^1^, Yusuke Niino^2^, Yuichi Hiraoka^3,4^, Kohichi Tanaka^4^, Atsushi

Miyawaki^2,5^, Johannes Hirrlinger^6,7^, Ko Matsui^1,8,*^

^1^Super-network Brain Physiology, Graduate School of Life Sciences, Tohoku University, Sendai 980-8577 Japan

^2^Laboratory for Cell Function Dynamics, RIKEN Center for Brain Science, Wako-city, Japan

^3^Laboratory of Molecular Neuroscience, Medical Research Institute (MRI), Tokyo Medical and Dental University

(TMDU) / Tokyo Institute of Technology, Tokyo, Japan

^4^Laboratory of Genome Editing for Biomedical Research, Medical Research Institute, Tokyo Medical and Dental

University (TMDU) / Tokyo Institute of Technology, Tokyo, Japan.

^5^Biotechnological Optics Research Team, RIKEN Center for Advanced Photonics, Wako-city, Japan

^6^Carl-Ludwig-Institute for Physiology, Faculty of Medicine, Leipzig University, Leipzig, Germany

^7^Department of Neurogenetics, Max-Planck-Institute for Multidisciplinary Sciences, Göttingen, Germany

^8^Super-network Brain Physiology, Graduate School of Medicine, Tohoku University, Sendai 980-8577 Japan

**Correspondence**

Ko Matsui, Super-network Brain Physiology, Graduate School of Life Sciences, Tohoku University, Sendai 980-8577

Japan.

Email: matsui@tohoku.ac.jp

**1** / **12**


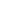

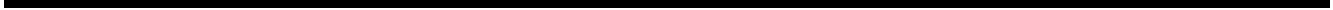

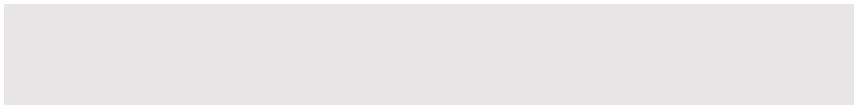


**Supplementary Discussions**

**Difference-metric photometry using FRET-based sensors**

Difference-metric photometry was initially developed for use with a FRET-based fluorescence

sensor for Ca^2+^ (YC_nano50_) in our previous publication (Ikoma, Sasaki, et al., 2023). In the current

study, we applied this methodology for the first time to another FRET-based fluorescence sensor,

ATeam, to evaluate the broader applicability of this analysis method. We then extended the

methodology to the newly created PYRS, with the consideration that, unlike ATeam, FRET

efficiency is reduced upon pyruvate binding to PYRS.

For Ca^2+^ or neurotransmitter concentration measurements, the majority of the research

community relies on GCaMP or GRAB-like sensors, which typically are employed with single-

wavelength fluorescence fluctuation analysis. Meanwhile, those working with FRET-based

sensors exclusively apply the ratio method. Through the following discussions, we aim to

emphasize the importance of multiwavelength fluorescence measurements and the necessity of

accounting for factors such as brain blood volume (BBV) and cytosolic pH, both of which can

influence fluorescence signals and are critical for accurately interpreting fluorescence fluctuations.

Consideration of these factors is particularly relevant for real-time analysis of *in vivo* biochemical

processes that occur on the seconds’ timescale.

In FRET-based recordings, the ratio of fYFP to fCFP is commonly used as an index of

concentration changes in the sensed molecule, in this case, ATP. However, it is important to note

that acidic pH can quench YFP more than CFP. As a result, a decrease in the fYFP/fCFP ratio

does not necessarily indicate a decrease in ATP levels. The pH sensitivity of YFP suggests that

cytosolic acidification may have occurred without a corresponding change in ATP levels.

Additionally, brain blood vessels, which do not express the artificially introduced fluorescence

sensors, appear as dark “shadows” in fluorescence imaging. Blood vessels are known to dilate or

constrict, resulting in changes in the dark (e.g., fluorescence-free) BBV within the field of view.

Such BBV changes are expected to influence all fluorescence signals (fCFP, fYFP, and dYFP)

from the brain parenchyma.

While fYFP is affected by changes in cytosolic ATP concentration, we assume that dYFP is

**2** / **12**

not, or at least to a much lesser extent. If we further assume that BBV changes and cytosolic pH

fluctuations affect both fYFP and dYFP equally, then the difference between fYFP and dYFP

(fYFP - dYFP) can be expected to provide a reliable indication of the ATP signal levels.

Consistent with these assumptions, fYFP and dYFP signal traces largely overlap under baseline

conditions, suggesting that BBV and pH fluctuations affect both signals similarly and that

neuronal cytosolic ATP fluctuations are minimal in calm, awake animals. However, during

epileptic neuronal hyperactivity, fYFP deviates significantly from dYFP, indicating that the

difference method (fYFP - dYFP) reliably reports changes in cytosolic ATP signal levels. Upon

sudden unexpected death in epilepsy (SUDEP), we observed a massive reduction in ATP signal

levels. This observation further confirms that the calculated ATP signal reliably reflects actual

cytosolic ATP changes, as ATP concentration is expected to decrease with the cessation of

cellular respiration and the death of the animal.

It is important to clarify that we do not claim the ATP signal calculated using the difference

method linearly reflects the actual cytosolic ATP concentration. We also do not have a means to

calibrate the ATP signal *in vivo*. Additionally, we need to assume that the fYFP signal follows a

monotonically increasing function of ATP concentration for the calculated ATP signal to

monotonically reflect the actual ATP concentration levels. We acknowledge that both the ratio

method (fYFP / dYFP) and the difference method (fYFP - dYFP) have limitations and rely on

several assumptions, as outlined above. We prefer the difference method because cytosolic pH

can fluctuate significantly under both physiological and pathophysiological conditions, as

observed with other probes specifically designed to measure cytosolic pH (Ikoma, Sasaki, et al.,

2023; Ikoma, Takahashi, et al., 2023; Tan et al., 2024). These pH fluctuations can be large enough

to substantially affect YFP fluorescence.

**Possibility of cytosolic pH fluctuations**

Although dYFP is inversely affected by changes in BBV, it is crucial to consider the potential

impact of cytosolic pH changes when interpreting dYFP fluctuations. As discussed earlier, YFP

fluorescence is highly sensitive to pH, with fluorescence decreasing upon acidification. A

prominent increase in dYFP was often observed in Thy1-ATeam mice during the induction of

**3** / **12**

epileptic neuronal hyperactivity in the early stages of kindling (Figure 3; but also see Figure S3).

This increase may reflect blood vessel constriction and/or neuronal cytosolic alkalization. A

transient increase in dYFP was also observed in Mlc1-tTA::tetO-PYRS mice, suggesting that

cytosolic alkalization of astrocytes may also occur during epileptic hyperactivity. A study using

a pH-sensitive fluorescence sensor has shown significant astrocytic cytosolic alkalization through

activation of the Na+-bicarbonate cotransporter (NBC) with epileptic hyperactivity in acute

hippocampal slice preparations (Onodera et al., 2021). To further confirm whether cytosolic pH

alkalization occurs in neurons and astrocytes in the hippocampus *in vivo* during the early stages

of epileptic kindling, a separate study using a pH-sensitive fluorescence sensor such as E^2^GFP

would be required (Ikoma, Sasaki, et al., 2023).

While it is generally assumed that endogenous buffers in the cytosol are sufficient to rapidly

equivalate pH under physiological conditions, multiple lines of evidence suggest that fluctuations

in cytosolic pH can occur *in vivo*. For example, it has been demonstrated that, in the lateral

hypothalamus, astrocytic cytosolic pH fluctuates during physiological REM sleep, as measured

with a fluorescence probe specifically designed to detect pH changes (Ikoma, Takahashi, et al.,

2023). Several pathways may lead to fluctuations in cytosolic pH in addition to NBC activation

(Deitmer, 1991; Pappas and Ransom, 1994; O’Connor et al., 1994). For instance, activation of

glutamate transporters induces H+ influx (Zerangue and Kavanaugh, 1996; Rose and Ransom,

1996), and the “non-selective” cation channels of NMDA receptors are known to permeate H+,

potentially leading to cytosolic acidification (Rathje et al., 2013). These cytosolic pH changes can

have several consequences, such as regulating astrocytic transmitter release through channels

(Beppu et al., 2014; Beppu et al., 2021) and influencing gap junction properties (Onodera et al.,

2021). Additionally, most biochemical and metabolic processes are sensitive to pH changes.

Given this evidence, it is highly likely that fluctuations in cytosolic pH play a role in cellular

signaling, and changes in pH could be significant enough to affect the YFP component of the

FRET-based sensors.

**Considering the possibility of local artifacts affecting fluorescence measurements**

Electrical stimulation can produce unintended local artifacts, such as slight movements in the

**4** / **12**

brain tissue surrounding the stimulation electrodes, which could lead to changes in the detected

fluorescence. To address this, we implanted an optical fiber in the hippocampus contralateral to

the stimulated hippocampus. In rodents, strong bilateral connections exist through the

hippocampal commissure (Amaral et al., 2007), and synchronized or spreading seizure activities

between the bilateral hippocampi have been observed (Wang et al., 2014; Shimoda et al., 2022).

As a result, unilateral hippocampal electrical stimulation should immediately induce epileptiform

activity in the contralateral hippocampus, allowing us to observe the metabolic effects of neuronal

hyperactivity without the potential confounding effects of local stimulation artifacts.

When the left hippocampus was stimulated, fluorescence changes were detected using the

optical fiber implanted in the right hippocampus of Thy1-ATeam mice (Figure S1). Similar to

ipsilateral stimulation, contralateral stimulation resulted in a positive shift in dYFP, suggesting

either a decrease in local BBV or neuronal cytosolic alkalinization during the epileptiform activity.

The ATP signal, calculated using the difference method (fYFP - dYFP), also showed a robust

decrease in neuronal cytosolic ATP signal, consistent with findings from ipsilateral stimulation.

Although bilateral recordings were performed in only a single mouse, no consistent differences

were observed between the two optical recording sites. As a result, the remainder of the current

study focused on fluorescence signals from the optical fiber ipsilateral to the stimulated

hippocampus.

**Possible bleaching effect of fluorophores**

To minimize fluorophore bleaching, the excitation light was delivered in short pulses. However,

it is still possible that continuous fiber photometry over the course of up to one week could result

in a reduction in the detected signal. A similar FRET fluorescence protein combination (CFP and

YFP) was used in a prior study from our lab (YC_nano50_; Ikoma, Sasaki et al., 2023) in kindling

experiments. In that study, an increase in the detected Ca^2+^ signal was observed, suggesting that

even with potential continuous photobleaching, a prominent signal increase can still be detected.

**Evaluation of the functionality of metabolite sensors**

As biochemical processes are expected to cease upon death, the ATP signal we measured

**5** / **12**

decreased dramatically during accidental sudden unexpected death in epilepsy (SUDEP)

encounters. Most previous studies using metabolite molecule sensors have lacked this final *in vivo*

validation of their functionality. Death events provide a valuable opportunity for such validation.

The three SUDEP incidents involving Thy1-ATeam animals observed in our study mostly

occurred during the early phase of the project. After this phase, we improved our understanding

of how to minimize SUDEP incidents, leading to fewer occurrences of these unfortunate events.

Consequently, we did not encounter any SUDEP incidents with astrocytic PYRS-expressing

animals. As lactate concentration increases with cessation of cellular respiration, a corresponding

rise in pyruvate might be expected.

Considering animal welfare, an overdose of anesthesia could be used as an alternative to the

rare SUDEP encounters. However, in our experience, death occurs very slowly under anesthesia,

and the fluorescence signal appears to be confounded by unknown factors, possibly including

brain edema. Cervical dislocation is another ethically approved option, but it likely causes

excessive movement of brain tissue. There is a need for methods to induce instantaneous

euthanasia that comply with animal welfare regulations. We predict that such methods will

become standardized in the future for all optical measurements of biomolecules in the brain, just

as ionomycin has become the standard for calibrating Ca^2+^ indicators *in situ*. At present, however,

accidental occurrences of SUDEP are the only observations that allow us to confirm the

functionality of our metabolite fluorescence probe *in vivo*.

**Possible mechanism of neuronal ATP recovery**

In the main manuscript, we primarily discussed the mechanisms underlying the reduction in

neuronal cytosolic ATP during epileptic AD generation. One possible explanation is that the

supply of energy-generating substrates to neurons becomes significantly limited during episodes

of epileptic hyperactivity. A rapid, transient increase in astrocytic pyruvate concentration was

observed during epileptic neuronal hyperactivity (Figure 5), potentially resulting from reduced

pyruvate efflux from astrocytes due to decreased MCT efficiency. These findings suggest that the

reduction in neuronal cytosolic ATP levels during hyperactivity could be partially attributed to

impaired transfer of pyruvate/lactate from astrocytes to neurons.

**6** / **12**

After a marked decrease in neuronal cytosolic ATP levels, ATP rapidly returned to baseline

within a couple of minutes. This recovery could occur as the inhibition of astrocyte-to-neuron

energy substrate transfer is alleviated. Alternatively, ATP production in neurons may be

upregulated after a delay following AD generation. Such a mechanism would allow the neuronal

metabolic system to meet ATP demands, prevent further decreases, and restore neuronal cytosolic

ATP back to its original baseline (Baeza-Lehnert et al., 2019).

**Possible presence of multiple energy supply routes**

It is possible that minor fluctuations in vessel diameter do not significantly affect neuronal

cytosolic ATP levels, as physiological mechanisms likely exist to maintain a constant net level of

neuronal cytosolic ATP. This stability could theoretically be achieved by providing neurons with

both high-efficiency and low-efficiency supply routes, both originating from a common blood

vessel source. If supply from this source diminishes slightly due to blood vessel constriction,

energy-generating substrates may be preferentially diverted to the high-efficiency route, ensuring

stable ATP levels. However, there may be a delay in adjusting the “valve” between these routes,

meaning small fluctuations in energy supply under physiological conditions could still transiently

affect neuronal cytosolic ATP levels. Future studies should investigate whether variations in

energy supply mechanisms are critical for the dynamic regulation of ATP levels.

**7** / **12**

**Supplementary References**

Amaral, D.G., Scharfman, H.E., Lavenex, P., 2007. The dentate gyrus: fundamental neuroanatomical

organization (dentate gyrus for dummies). Prog. Brain. Res. 163, 3–22.

https://doi.org/10.1016/S0079-6123(07)63001-5.

Baeza-Lehnert, F., Saab, A.S., Gutiérrez, R., Larenas, V., Díaz, E., Horn, M., Vargas, M., Hösli, L.,

Stobart, J., Hirrlinger, J., Weber, B., Barros, L.F., 2019. Non-Canonical Control of Neuronal

Energy Status by the Na+ Pump. Cell. Metab. 29, 668-680.e4.

https://doi.org/10.1016/j.cmet.2018.11.005.

Beppu, K., Kubo, N., Matsui, K., 2021. Glial amplification of synaptic signals. J. Physiol. 599, 2085–

2102. https://doi.org/10.1113/JP280857.

Beppu, K., Sasaki, T., Tanaka, K.F., Yamanaka, A., Fukazawa, Y., Shigemoto, R., Matsui, K., 2014.

Optogenetic countering of glial acidosis suppresses glial glutamate release and ischemic brain

damage. Neuron. 81, 314–320. https://doi.org/10.1016/j.neuron.2013.11.011.

Deitmer, J.W., 1991. Electrogenic sodium-dependent bicarbonate secretion by glial cells of the leech

central nervous system. J. Gen. Physiol. 98, 637–655. https://doi.org/10.1085/jgp.98.3.637.

Ikoma, Y., Sasaki, D., Matsui, K., 2023. Local brain environment changes associated with

epileptogenesis. Brain. 146, 576–586. https://doi.org/10.1093/brain/awac355.

Ikoma, Y., Takahashi, Y., Sasaki, D., Matsui, K., 2023. Properties of REM sleep alterations with

epilepsy. Brain. 146, 2431–2442. https://doi.org/10.1093/brain/awac499.

O'Connor, E.R., Sontheimer, H., Ransom, B.R., 1994. Rat hippocampal astrocytes exhibit electrogenic

sodium-bicarbonate co-transport. J. Neurophysiol. 72, 2580–2589.

https://doi.org/10.1152/jn.1994.72.6.2580.

Onodera, M., Meyer, J., Furukawa, K., Hiraoka, Y., Aida, T., Tanaka, K., Tanaka, K.F., Rose, C.R.,

Matsui, K., 2021. Exacerbation of epilepsy by astrocyte alkalization and gap junction uncoupling. J.

Neurosci. 41, 2106–2118. https://doi.org/10.1523/JNEUROSCI.2365-20.2020.

Pappas, C.A., Ransom, B.R., 1994. Depolarization-induced alkalinization (DIA) in rat hippocampal

astrocytes. J. Neurophysiol. 72, 2816–2826. https://doi.org/10.1152/jn.1994.72.6.2816.

Rathje, M., Fang, H., Bachman, J.L., Anggono, V., Gether, U., Huganir, R.L., Madsen, K.L., 2013.

AMPA receptor pHluorin-GluA2 reports NMDA receptor-induced intracellular acidification in

hippocampal neurons. Proc. Natl. Acad. Sci. U. S. A. 110, 14426–14431.

https://doi.org/10.1073/pnas.1312982110.

Rose, C.R., Ransom, B.R., 1996. Mechanisms of H+ and Na+ changes induced by glutamate, kainate, and

D-aspartate in rat hippocampal astrocytes. J. Neurosci. 16, 5393–5404.

https://doi.org/10.1523/jneurosci.16-17-05393.1996.

Shimoda, Y., Beppu, K., Ikoma, Y., Morizawa, Y.M., Zuguchi, S., Hino, U., Yano, R., Sugiura, Y.,

Moritoh, S., Fukazawa, Y., Suematsu, M., Mushiake, H., Nakasato, N., Iwasaki, M., Tanaka, K.F.,

Tominaga, T., Matsui, K., 2022. Optogenetic stimulus-triggered acquisition of seizure resistance.

Neurobiol. Dis. 163, 105602. https://doi.org/10.1016/j.nbd.2021.105602.

Tan, W., Ikoma, Y., Takahashi, Y., Konno, A., Hirai, H., Hirase, H., Matsui, K., 2024. Anxiety control by

astrocytes in the lateral habenula. Neurosci. Res. 205, 1–15.

https://doi.org/10.1016/j.neures.2024.01.006.

Wang, Y., Toprani, S., Tang, Y., Vrabec, T., Durand, D.M., 2014. Mechanism of highly synchronized

bilateral hippocampal activity. Exp. Neurol. 251, 101–111.

https://doi.org/10.1016/j.expneurol.2013.11.014.

Zerangue, N., Kavanaugh, M.P., 1996. Flux coupling in a neuronal glutamate transporter. Nature. 383,

634–637. https://doi.org/10.1038/383634a0.

**8** / **12**

**Supplementary Figures**

**Supplementary Figure 1.** Neuronal cytosolic ATP reduction was also observed in the

contralateral hippocampus. (A) The positions of the EEG recording screw electrode, ground screw

electrode, optical fiber accompanied with paired stimulus electrodes (Fluo Rec1), and an

additional optical fiber dedicated to recording the fluorescence signal from the contralateral right

hippocampus (Fluo Rec2) are illustrated. The latter optical fiber recorded from the side opposite

the electrically stimulated left hippocampus. (B) A fluorescent image shows the placement of the

optical fibers in the hippocampus of a Thy1-ATeam transgenic mouse, with the right optical fiber

used for recording only and the left optical fiber paired with stimulus electrodes. (C) The left

hippocampus was electrically stimulated, and fluorescence signal traces from the right

contralateral hippocampus (the side opposite to the stimulation) are shown, displaying

fluctuations in fCFP (blue), dYFP (red), and fYFP (green) fluorescence. The fluorescence signals

fluctuated similarly to those recorded from the ipsilateral side, and neuronal cytosolic ATP signal

reduction was also observed in the contralateral hippocampus, with no apparent differences

between the two sides. (D) Fluorescence signal traces recorded from the ipsilateral hippocampus

(left side) in the same animal as in panel C.

**9** / **12**


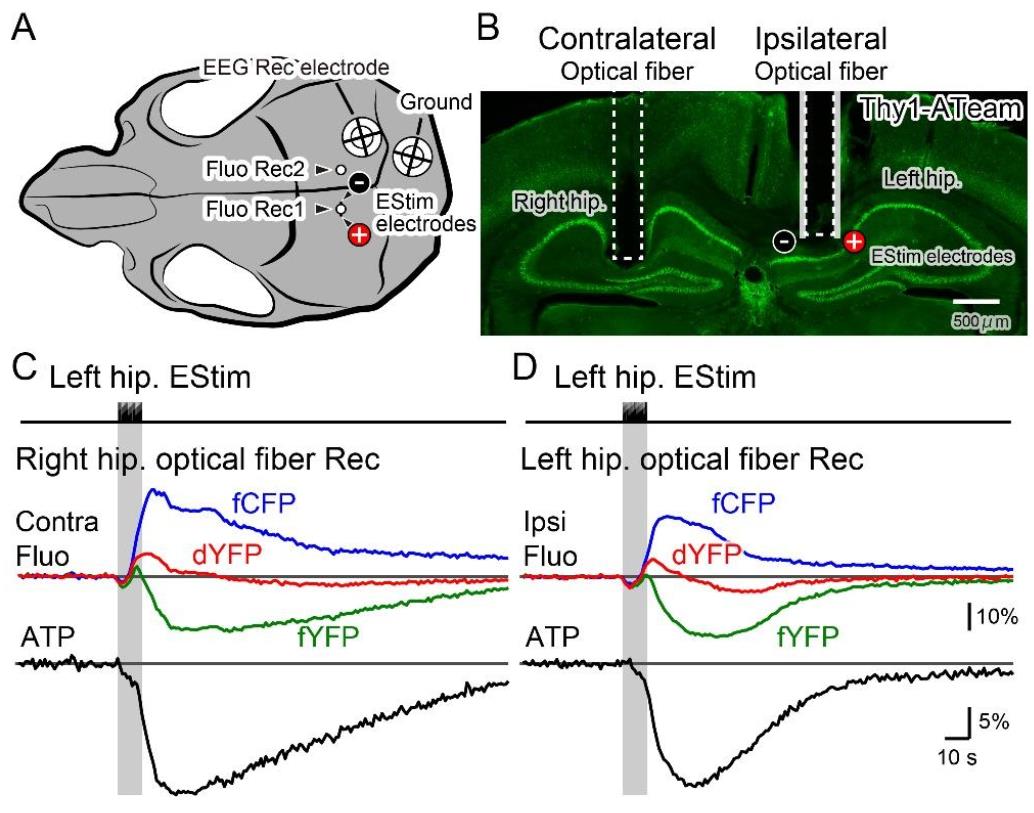


**Supplementary Figure 2.** Comparison of astrocytic pyruvate and neuronal ATP signal dynamics.

Energy molecule dynamics during three episodes of neuronal hyperactivity were examined in

Mlc1-tTA::tetO-PYRS and Thy1-ATeam mice (representative data from n = 1 and 1 animals,

respectively). Summarized data from multiple animals are presented in Figures 5E and 5F. (A)

The onset of the first phase of the pyruvate signal increase in astrocytes (top, 3.25 ± 0.04, n = 3

episodes) and the onset of the ATP signal reduction in neurons (bottom, 12.67 ± 0.88, n = 3

episodes) are shown. (B) The initial positive peak of the pyruvate signal in astrocytes corresponds

to the onset of its subsequent decrease (top, 19.97 ± 2.89 s, n = 3 episodes). The time of the

negative peak of the ATP signal in neurons is also shown (bottom, 34.00 ± 3.21 s, n = 3 episodes).

Individual data points are presented alongside the mean ± s.e.m.

**10** / **12**


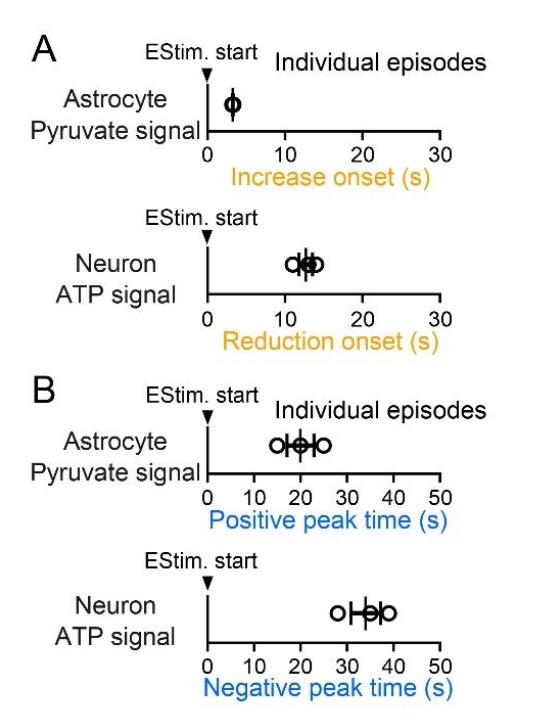


**Supplementary Figure 3.** Absence of dYFP increase during epileptic neuronal hyperactivity in

a Thy1-ATeam mouse. Typically, a transient dYFP increase occurs immediately following

hippocampal electrical stimulation in the early stages of kindling (see Figure 6). This increase

may be associated with blood vessel constriction leading to reduced BBV, or with neuronal

cytosolic alkalinization. However, with continued epileptic kindling, this positive deflection of

the dYFP signal generally attenuates. In the example shown here, no detectable dYFP increase

was observed, even during the early stage of kindling. EEG traces shown on top were band-pass

filtered at 1 - 100 Hz.

**11** / **12**


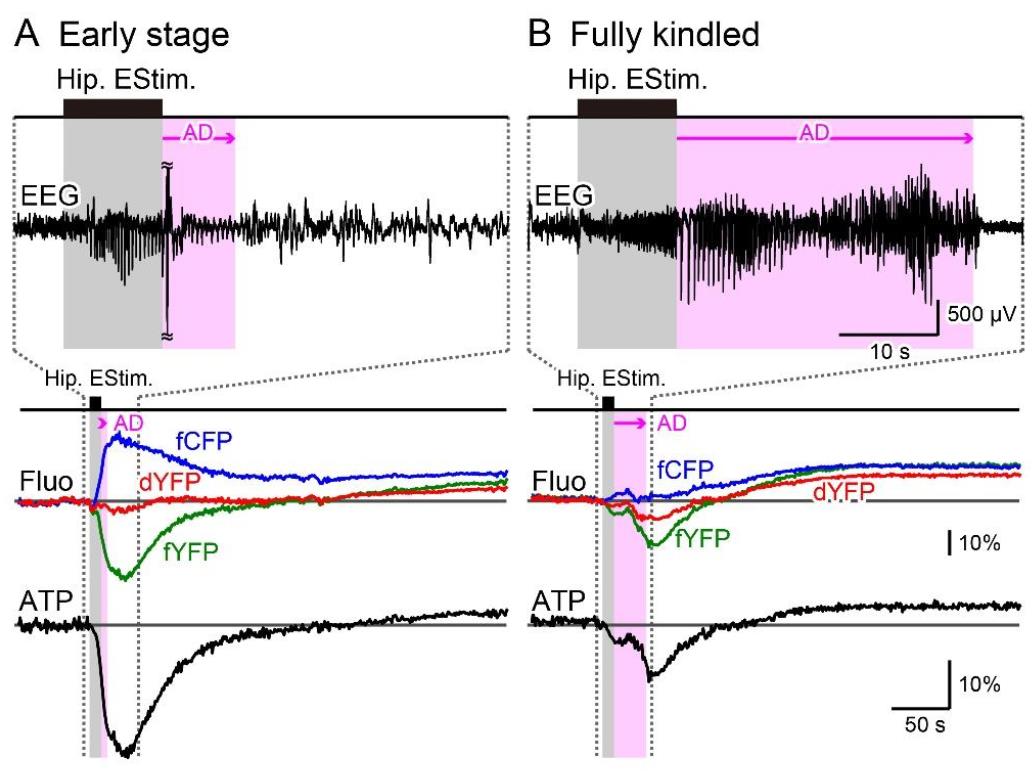


**Supplementary Figure 4.** Integration of Texas Red signal up to AD cessation time. (A) In Figure

7, the Texas Red signal was integrated from the start of electrical stimulation to the time of ATP

signal’s negative peak. In this figure, the Texas Red signal was instead integrated up to the AD

cessation time. (B) The cumulative Texas Red fluorescence was consistently positive and higher

in the Fully kindled stage compared to the Early stage; however, this difference was not

statistically significant (Early stage 64.36 ± 36.65 a.u., Fully kindled 851.24 ± 242.20 a.u., n = 3

animals, paired t-test, degree of freedom = 2, t = -3.597, p = 0.069). Individual data points are

presented alongside the mean ± s.e.m. See Figure 7B for comparison. (C) The relationship

between ATP signal reduction and cumulative Texas Red fluorescence is plotted. Cumulative

Texas Red consistently increased with kindling; however, the magnitude of ATP signal reduction

decreased from the Early stage (blue dot) to the Fully kindled stage (red dot) within the same

animal. Data pairs from the same animal are connected by straight dashed lines.

**12** / **12**


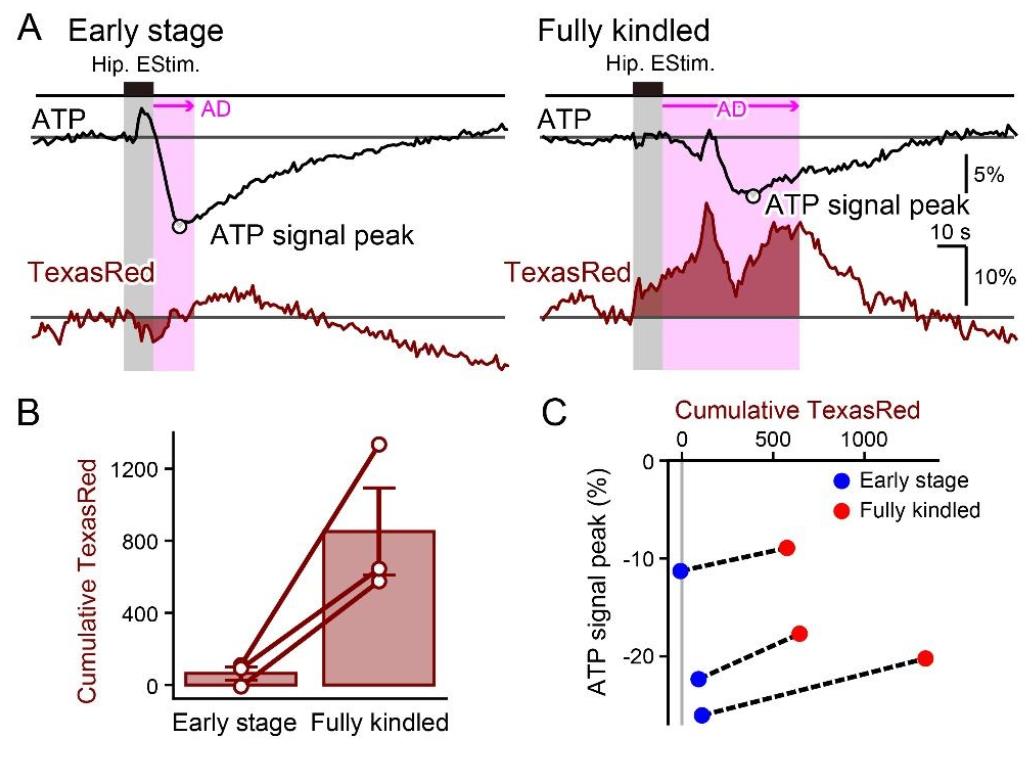

Supplement: Supplementary file 1 — Data S1. Supporting Information. [file JNC-169-0-s001.docx]
